# Supplementary material for: A non-Hermitian optical atomic mirror
Source: Nat Commun. 2022 Aug 6;13:4598. doi: 10.1038/s41467-022-32372-3 (PMC9357005; doi:10.1038/s41467-022-32372-3)
Supplement: Supplementary file 1 — Supplementary Information for “A non-Hermitian optical atomic mirror” [file 41467_2022_32372_MOESM1_ESM.pdf]

Supplementary Information for "A non-Hermitian optical atomic mirror"

Yi-Cheng Wang,[1, 2, *] Jhih-Shih You,[3, †] and H. H. Jen[2, ‡]
[1]Department of Physics, National Taiwan University, Taipei 10617, Taiwan
[2]Institute of Atomic and Molecular Sciences, Academia Sinica, Taipei 10617, Taiwan
[3]Department of Physics, National Taiwan Normal University, Taipei 11677, Taiwan

The supplementary information is organized as follows. Note 1 reviews the general formalism of a two-dimensional (2D) atomic array in free space. Note 2 presents the model-independent generalization of Euler-Maclaurin formula we develop to calculate the infinite summation in the bulk spectrum. In notes 3-5, we discusses the 2D atomic lattices that extend infinitely in two directions, atomic lattices in ribbon geometries, and finite-size 2D atomic lattices, respectively. Finally, note 6 presents the optical responses of realistic finite atomic arrays.

SUPPLEMENTARY NOTE 1. OPTICAL ATOMIC MIRROR

1. General formalism

We first consider a coupled singly-excited of two-dimensional (2D) atomic lattice in free space. Each atom is a four-level system formed by one ground state $|g\rangle$ and three degenerate excited states $|x\rangle$, $|y\rangle$, and $|z\rangle$ with resonant frequency ω_0 and decay rate Γ_0. Within the dipole and Markov approximations, we eliminate the reservoir photons in light-matter interactions, and the N-atom non-Hermitian effective Hamiltonian is given by [1]

$$\hat{\mathcal{H}}_{\rm eff}=\sum_{m=1}^{N}\sum_{\nu=x,y,z}\hbar\left(\omega_0-i\frac{\Gamma_0}{2}\right)\hat{\sigma}^\dagger_{m\nu}\hat{\sigma}_{m\nu} -\frac{3\pi\hbar\Gamma_0 c}{\omega_0}\sum_{m\neq n}\sum_{\mu,\nu=x,y,z}G_{\mu\nu}(\mathbf{r}_m-\mathbf{r}_n)\hat{\sigma}^\dagger_{m\mu}\hat{\sigma}_{n\nu}, \tag{1}$$

where $\hat{\sigma}_{n\nu}=|g_n\rangle\langle\nu_n|$ is the lowering operator of nth atom and $G_{\mu\nu}(\mathbf{r})$ is the dyadic Green's function representing the resonant dipole-dipole interaction (RDDI) between two atoms separated by a distance $\mathbf{r}$ with dipoles aligned along μ and ν directions. For an infinite periodic 2D Bravais lattice, single excitation can be well described in reciprocal space, and the eigenstates of Eq. (1) are Bloch states according to the translational symmetry. The Fourier transformations of atomic operators is defined as

$$\hat{\sigma}_{n\nu}=\sum_{\mathbf{k}}e^{i\mathbf{k}\cdot\mathbf{r}_n}\hat{\sigma}_{\mathbf{k}\nu}, \tag{2}$$

where the sum over wave vector $\mathbf{k}$ is restricted to the first Brillouin zone (BZ). Defining the Bloch state with a quasimomentum $\mathbf{k}_{\rm B}$ in the Cartesian basis, $|\psi_{\mathbf{k}_{\rm B}}\rangle=\left(c_x\hat{\sigma}^\dagger_{\mathbf{k}_{\rm B}x},c_y\hat{\sigma}^\dagger_{\mathbf{k}_{\rm B}y},c_z\hat{\sigma}^\dagger_{\mathbf{k}_{\rm B}z}\right)^T|g\rangle^{\otimes N\to\infty}$, the eigenvalue problem of Eq. (1) reads

$$E_{\mathbf{k}_{\rm B}}=\langle\psi_{\mathbf{k}_{\rm B}}|\hat{\mathcal{H}}_{\rm eff}|\psi_{\mathbf{k}_{\rm B}}\rangle=\sum_{\nu}\hbar\left(\omega_0-i\frac{\Gamma_0}{2}\right)c^*_\nu c_\nu -\frac{3\pi\hbar\Gamma_0 c}{\omega_0}\sum_{\mu,\nu}\sum_{\mathbf{R}\neq 0}e^{-i\mathbf{k}_{\rm B}\cdot\mathbf{R}}G_{\mu\nu}(\mathbf{R})c^*_\mu c_\nu, \tag{3}$$

where $\mathbf{R}$ is real space lattice vectors. Equation (3) is the same as diagonalization of a 3×3 matrix and we can use it to define the Hamiltonian kernel $\mathcal{H}_{\rm eff}(\mathbf{k}_{\rm B})$. The challenge in the determination of dispersion relation described in Eq. (3) is the calculation on 2D Fourier transformation of dyadic Green's function since it's an infinite summation with oscillatory terms. The standard procedure to deal with this infinite sum is Ewald's method [2], which computes the slowly convergent part in reciprocal space to avoid the oscillatory terms in real space. However, an artificial truncation or the ultraviolet frequency cutoff [1] is necessary to reach the desired precision. Here, we exploit the 2D generalization of Euler-Maclaurin formula to give a model-independent approach to calculate the infinite sum in reciprocal space without the ultraviolet frequency cutoff, and the truncation is determined by a self-consistent way.

2. Atomic lattices in free space

Dyadic Green's function describing the resonant dipole-dipole interaction in free space at resonant frequency ω has the following form:

$$\overline{\overline{\mathbf{G}}}_0(\mathbf{r})=\frac{e^{iqr}}{4\pi r}\left[\left(1+\frac{iqr-1}{q^2r^2}\right)\overline{\overline{\mathbf{I}}} +\left(-1+\frac{3-3iqr}{q^2r^2}\right)\frac{\mathbf{r}\otimes\mathbf{r}}{r^2}\right], \tag{4}$$

where $q=\omega/c$ and $r=|\mathbf{r}|$ is the distance between two dipole emitters, and this can be obtained either from classical electromagnetism or quantum optical treatment [3]. Because we are interested in the closed form of dyadic Green's function in reciprocal space, we use Weyl decomposition and integrate out the momentum perpendicular

* r09222006@ntu.edu.tw
† jhihshihyou@gmail.com
‡ sappyjen@gmail.com

to 2D atomic lattice placed in the xy-plane to rewrite Eq. (4) as

$$\overline{\overline{\mathbf{G}}}_0(\mathbf{r}_\parallel) = \int \frac{d^2k_\parallel}{(2\pi)^3}\left[\overline{\overline{\mathbf{I}}} - \left(1-\frac{\mathbf{k}_\parallel^2}{q^2}\right)\mathbf{e}_z\otimes\mathbf{e}_z - \frac{1}{q^2}\mathbf{k}_\parallel\otimes\mathbf{k}_\parallel\right] \times\left[\frac{i\pi\Theta\left(q^2-\mathbf{k}_\parallel^2\right)}{\sqrt{q^2-\mathbf{k}_\parallel^2}} + \frac{\pi\Theta\left(\mathbf{k}_\parallel^2-q^2\right)}{\sqrt{\mathbf{k}_\parallel^2-q^2}}\right]e^{i\mathbf{k}_\parallel\cdot\mathbf{r}_\parallel} = \int\frac{d^2k_\parallel}{(2\pi)^2}e^{i\mathbf{k}_\parallel\cdot\mathbf{r}_\parallel}\overline{\overline{\mathbf{g}}}_0(\mathbf{k}_\parallel), \tag{5}$$

where the integrand $\overline{\overline{\mathbf{g}}}_0(\mathbf{k}_\parallel)$ is the dyadic Green's function in reciprocal space and Θ denotes the Heaviside step function. By inserting Eq. (5) into Eq. (3) and using Poisson's identity, the effective Hamiltonian kernel for infinite 2D Bravais lattice $\{\mathbf{R}\}$ with two lattice vectors $\mathbf{a}_1$ and $\mathbf{a}_2$ is given by

$$\mathcal{H}_{\rm eff}^{\rm 3D}(\mathbf{k}_{\rm B}) = \hbar\left(\omega_0 - i\frac{\Gamma_0}{2}\right)\overline{\overline{\mathbf{I}}} - \frac{3\pi\hbar\Gamma_0 c}{\omega_0}\left[\overline{\overline{\mathbf{S}}}_0(\mathbf{k}_{\rm B}) - \overline{\overline{\mathbf{G}}}_0(\mathbf{0})\right], \tag{6}$$

$$\overline{\overline{\mathbf{S}}}_0(\mathbf{k}_{\rm B}) = \sum_{\mathbf{G}}\frac{\overline{\overline{\mathbf{g}}}_0(\mathbf{G}+\mathbf{k}_{\rm B})}{|\mathbf{a}_1\times\mathbf{a}_2|}, \tag{7}$$

$$\sum_{\mathbf{R}\neq\mathbf{0}} e^{-i\mathbf{k}_{\rm B}\cdot\mathbf{R}}\overline{\overline{\mathbf{G}}}_0(\mathbf{R}) = \overline{\overline{\mathbf{S}}}_0(\mathbf{k}_{\rm B}) - \overline{\overline{\mathbf{G}}}_0(\mathbf{0}) = \left[\sum_{\mathbf{G}} - \int\frac{|\mathbf{a}_1\times\mathbf{a}_2|}{(2\pi)^2}d^2G\right]\frac{\overline{\overline{\mathbf{g}}}_0(\mathbf{G}+\mathbf{k}_{\rm B})}{|\mathbf{a}_1\times\mathbf{a}_2|}, \tag{8}$$

where the summation in Eq. (6) runs over all reciprocal lattice vectors $\mathbf{G}$. As we can see from Eq. (5), the infinite periodic sum $\overline{\overline{\mathbf{S}}}_0(\mathbf{k}_{\rm B})$ in Eq. (7) describing how atoms in Bravais lattice $\{\mathbf{R}\}$ are affected by all atoms (including the self-interaction) contains no oscillatory terms and diverges. While $\overline{\overline{\mathbf{G}}}_0(\mathbf{0})$ as the self-interaction is also divergent according to Eq. (4), the difference between the interactions of all atoms $\overline{\overline{\mathbf{S}}}_0(\mathbf{k}_{\rm B})$ and $\overline{\overline{\mathbf{G}}}_0(\mathbf{0})$ in Eq. (8), which describes how atoms in Bravais lattice $\{\mathbf{R}\}$ are affected by the other atoms (excluding the self-interaction), is physically meaningful and convergent, and it can be calculated via the 2D generalization of Euler-Maclaurin formula since it is in the form of the difference between the summation and the integration of the same function. One of the essences of Euler-Macularin formula is to approximate this difference over a period by the difference of higher-order derivatives at the boundaries, such that the difference within any duplication of the periodic region can be viewed as the difference of higher-order derivatives at larger boundaries. Once we push one of the boundaries to infinity in reciprocal space, the presence of ultraviolet frequency cutoff ensures that high frequency terms would not contribute to the low energy physics, and we can simply approximate Eq. (8) out of certain boundary ∂R by higher-order derivatives of dyadic Green's function in reciprocal space $\overline{\overline{\mathbf{g}}}_0(\mathbf{G}+\mathbf{k}_{\rm B})$ at this boundary.

For a non-Hermitian effective Hamiltonian given by Eq. (6), the first term describes the single atom with a decay rate Γ_0 and the second term involves the collective RDDI without the self-interaction, such that the real and imaginary parts of dyadic Green's function correspond to the collective Lamb shift and collective decay rate. According to Eq. (5), only $\overline{\overline{\mathbf{g}}}_0(\mathbf{G}+\mathbf{k}_{\rm B})$ is responsible for the collective decay when $\mathbf{G}+\mathbf{k}_{\rm B}$ lies within the light cone defined by $|\mathbf{G}+\mathbf{k}_{\rm B}|\leq q$, since the imaginary part of self-interaction $\overline{\overline{\mathbf{G}}}_0(\mathbf{0})$ cancels the single atom decay rate Γ_0. That is, this system becomes Hermitian again if $\mathbf{G}+\mathbf{k}_{\rm B}$ lies out of the light cone for all reciprocal lattice vectors $\mathbf{G}$, and the corresponding Bloch state is dissipationless and lives, in principle, forever in the atomic lattice.

The existence of light cone can be realized from the elimination of out-of-plane momentum in Eq. (5):

$$\overline{\overline{\mathbf{G}}}_0(\mathbf{r}) = \int\frac{d^2k_\parallel}{(2\pi)^3}e^{i\mathbf{k}_\parallel\cdot\mathbf{r}_\parallel}\int dk_z e^{ik_z z}\frac{\overline{\overline{\mathbf{I}}} - \frac{1}{q^2}\mathbf{k}\otimes\mathbf{k}}{\mathbf{k}_\parallel^2 + k_z^2 - q^2 - i\epsilon}, \tag{9}$$

where ϵ is an infinitesimal positive value to ensure the causality. The poles on complex k_z plane are given by $\pm[\sqrt{q^2-\mathbf{k}_\parallel^2}+i\epsilon]\Theta(q^2-\mathbf{k}_\parallel^2)$ and $\pm[i\sqrt{\mathbf{k}_\parallel^2-q^2}+\epsilon]\Theta(\mathbf{k}_\parallel^2-q^2)$. These respective on-shell momenta, as the real photon exchange process in RDDI, relate to the outgoing field propagating out of atomic lattice with a real out-of-plane momentum and evanescent wave localized in atomic lattice due to the exponentially decay in out-of-plane direction. It's clear to see that only those modes lie within the light cone contribute to the collective decay and the presence of these radiative eigenstates may lead to the radiative edge modes in finite system [4].

3. *Bravais and non-Bravais lattices*

Although we only discuss the Bravais lattice in the main text, here we briefly review the formalism of effective Hamiltonian kernel for a non-Bravais lattice in order to demonstrate that our 2D generalization of Euler-Maclaurin formula can be used in non-Bravais lattices. Here we take the Kagome lattice for example. Kagome lattice, as a non-Bravais lattice composed of three Bravais triangular sublattices, has three inequivalent sites denoted by 1, 2, and 3, then the Bloch state becomes a 9×1 column vector defined in the basis of the tensor product of sublattices and polarizations $|\psi_{\mathbf{k}_{\rm B}}\rangle = (\,|\psi_{\mathbf{k}_{\rm B}}^{(1)}\rangle,\ |\psi_{\mathbf{k}_{\rm B}}^{(2)}\rangle,\ |\psi_{\mathbf{k}_{\rm B}}^{(3)}\rangle\,)^T$, where $|\psi_{\mathbf{k}_{\rm B}}^{(i)}\rangle = (c_x^{(i)}\hat{\sigma}_{\mathbf{k}_{\rm B}x}^{(i)\dagger},\ c_y^{(i)}\hat{\sigma}_{\mathbf{k}_{\rm B}y}^{(i)\dagger},\ c_z^{(i)}\hat{\sigma}_{\mathbf{k}_{\rm B}z}^{(i)\dagger})^T\,|g\rangle^{\otimes N\to\infty}$. In this case, the 9×9 effective Hamiltonian kernel for a 2D Kagome lattice reads

$$\mathcal{H}_{\text{eff}}\left(\mathbf{k}_{\mathrm{B}}\right)=\hbar\left(\omega_0-i\frac{\Gamma_0}{2}\right)\begin{pmatrix}\overline{\overline{\mathbf{I}}} & 0 & 0\\ 0 & \overline{\overline{\mathbf{I}}} & 0\\ 0 & 0 & \overline{\overline{\mathbf{I}}}\end{pmatrix} \tag{10}$$
$$-\frac{3\pi\hbar\Gamma_0 c}{\omega_0}\begin{pmatrix}\sum\limits_{\mathbf{R}\neq0}e^{-i\mathbf{k}_{\mathrm{B}}\cdot\mathbf{R}}\overline{\overline{\mathbf{G}}}_0(\mathbf{R}) & \sum\limits_{\mathbf{R}}e^{-i\mathbf{k}_{\mathrm{B}}\cdot(\mathbf{R}+\mathbf{b}_{12})}\overline{\overline{\mathbf{G}}}_0(\mathbf{R}+\mathbf{b}_{12}) & \sum\limits_{\mathbf{R}}e^{-i\mathbf{k}_{\mathrm{B}}\cdot(\mathbf{R}+\mathbf{b}_{13})}\overline{\overline{\mathbf{G}}}_0(\mathbf{R}+\mathbf{b}_{13})\\ \sum\limits_{\mathbf{R}}e^{-i\mathbf{k}_{\mathrm{B}}\cdot(\mathbf{R}+\mathbf{b}_{21})}\overline{\overline{\mathbf{G}}}_0(\mathbf{R}+\mathbf{b}_{21}) & \sum\limits_{\mathbf{R}\neq0}e^{-i\mathbf{k}_{\mathrm{B}}\cdot\mathbf{R}}\overline{\overline{\mathbf{G}}}_0(\mathbf{R}) & \sum\limits_{\mathbf{R}}e^{-i\mathbf{k}_{\mathrm{B}}\cdot(\mathbf{R}+\mathbf{b}_{23})}\overline{\overline{\mathbf{G}}}_0(\mathbf{R}+\mathbf{b}_{23})\\ \sum\limits_{\mathbf{R}}e^{-i\mathbf{k}_{\mathrm{B}}\cdot(\mathbf{R}+\mathbf{b}_{31})}\overline{\overline{\mathbf{G}}}_0(\mathbf{R}+\mathbf{b}_{31}) & \sum\limits_{\mathbf{R}}e^{-i\mathbf{k}_{\mathrm{B}}\cdot(\mathbf{R}+\mathbf{b}_{32})}\overline{\overline{\mathbf{G}}}_0(\mathbf{R}+\mathbf{b}_{32}) & \sum\limits_{\mathbf{R}\neq0}e^{-i\mathbf{k}_{\mathrm{B}}\cdot\mathbf{R}}\overline{\overline{\mathbf{G}}}_0(\mathbf{R})\end{pmatrix}.$$

The block diagonal part of Eq. (10) shows the effective Hamiltonian of each triangular sublattice and its RDDI excludes the self-interaction, while the block off-diagonal term describing the RDDI between two sublattices keeps the whole contribution from all triangular lattice vectors $\mathbf{R}$. For simplicity, we focus on in-plane polarizations since it is decoupled from out-of-plane polarization for 2D geometry according to Eq. (5), from which we obtain their corresponding photonic band structures in the main text.

SUPPLEMENTARY NOTE 2. EULER-MACLAURIN FORMULA

1. 2D generalization of Euler-Maclaurin formula

We use Bernoulli polynomials $B_n(x)$ and Bernoulli numbers B_n [5] to derive the 2D generalization of Euler-Maclaurin formula using a similar procedure in the one-dimensional (1D) case. Starting from the summation of a function $f(x,y)$ over 2D rectangular lattice points $\mathbf{R}=mb_1\mathbf{e}_x+nb_2\mathbf{e}_y$, which is in the form of the linear combination of two independent lattice vectors $\mathbf{b}_1=b_1\mathbf{e}_x$ and $\mathbf{b}_2=b_2\mathbf{e}_y$, and the integral of $f(x,y)$ over a minimal rectangular period $S_{\square}=\{(x,y)|\,k\le x\le k+b_1,\, l\le y\le l+b_2\}$ can be written as (assume $f(x,y)$ is a smooth function)

$$\begin{aligned}
&\int_l^{l+b_2}dy\int_k^{k+b_1}dxf\left(x,y\right)=\int_l^{l+b_2}dy\left\{b_1B_1(\tfrac{x-k}{b_1})f(x,y)-\sum_{i=1}^{\lfloor M_x/2\rfloor}\frac{b_1^{2i}}{(2i)!}B_{2i}(\tfrac{x-k}{b_1})\partial_x^{2i-1}f(x,y)\right]\Bigg|_k^{k+b_1}+R_{M_x}\Bigg\}\\
=&\left\{b_1B_1(\tfrac{x-k}{b_1})\left[b_2B_1(\tfrac{y-l}{b_2})f(x,y)-\sum_{j=1}^{\lfloor M_y/2\rfloor}\frac{b_2^{2j}}{(2j)!}B_{2j}(\tfrac{y-l}{b_2})\partial_y^{2j-1}f(x,y)\right]\right\}\Bigg|_k^{k+b_1}\Bigg|_l^{l+b_2}\\
&-\left\{\sum_{i=1}^{\lfloor M_x/2\rfloor}\frac{b_1^{2i}}{(2i)!}B_{2i}(\tfrac{x-k}{b_1})\left[b_2B_1(\tfrac{y-l}{b_2})\partial_x^{2i-1}f(x,y)-\sum_{j=1}^{\lfloor M_y/2\rfloor}\frac{b_2^{2j}}{(2j)!}B_{2j}(\tfrac{y-l}{b_2})\partial_x^{2i-1}\partial_y^{2j-1}f(x,y)\right]\right\}\Bigg|_k^{k+b_2}\Bigg|_l^{l+b_1}+R_{M_x,M_y}\\
=&\frac{1}{4}b_1b_2\left[f_{k+b_1,l+b_2}+f_{k+b_1,l}+f_{k,l+b_2}+f_{k,l}\right]\\
&-\frac{b_2}{2}\sum_{i=1}^{\lfloor M_x/2\rfloor}\frac{b_1^{2i}}{(2i)!}B_{2i}\left[\partial_x^{2i-1}f_{k+b_1,l+b_2}+\partial_x^{2i-1}f_{k+b_1,l}-\partial_x^{2i-1}f_{k,l+b_2}-\partial_x^{2i-1}f_{k,l}\right]\\
&-\frac{b_1}{2}\sum_{j=1}^{\lfloor M_y/2\rfloor}\frac{b_2^{2j}}{(2j)!}B_{2j}\left[\partial_y^{2j-1}f_{k+b_1,l+b_2}+\partial_y^{2j-1}f_{k,l+b_2}-\partial_y^{2j-1}f_{k+b_1,l}-\partial_y^{2j-1}f_{k,l}\right]\\
&+\sum_{i=1}^{\lfloor M_x/2\rfloor}\sum_{j=1}^{\lfloor M_y/2\rfloor}\frac{b_1^{2i}}{(2i)!}\frac{b_2^{2j}}{(2j)!}B_{2i}B_{2j}\left[\partial_x^{2i-1}\partial_y^{2j-1}f_{k+b_1,l+b_2}-\partial_x^{2i-1}\partial_y^{2j-1}f_{k+b_1,l}-\partial_x^{2i-1}\partial_y^{2j-1}f_{k,l+b_2}+\partial_x^{2i-1}\partial_y^{2j-1}f_{k,l}\right]\\
&+R_{M_x,M_y}.
\end{aligned} \tag{11}$$

The first equal sign in the above is nothing but 1D Euler-Maclaurin formula expanded to the M_xth order, where R_{M_x} is the remainder term. Since x and y coordinates are linearly independent, we directly obtain the second equal sign by treating it as the 1D case. The last equal sign is our 2D generalization of Euler-Maclaurin formula expanded to the M_xth order in x and the M_yth order in y for 2D rectangular lattice points. Note that this formula respects the symmetry in the interchange of (x,k,b_1) and (y,l,b_2), which is important for the periodic summation.

For 2D triangular lattice points $\mathbf{R}=m\mathbf{b}_1+n\mathbf{b}_2=\frac{1}{2}b(m+n)\mathbf{e}_x+\frac{\sqrt{3}}{2}b(-m+n)\mathbf{e}_y$, two lattice vectors are not orthogonal such that we can not directly follow the procedure in Eq. (11). We try to separate the integral over a minimal triangular period $S_\triangle=\{(x,y)|\,y\geq l,\ \sqrt{3}(x-k)-(y-l)\geq 0,\ \sqrt{3}(x-k)+(y-l)\leq\sqrt{3}b\}$, i.e., an equilateral triangle with vertices (k,l), $(k+b,l)$ and $(k+\frac{1}{2}b,l+\frac{\sqrt{3}}{2}b)$, into the following two parts

$$
\begin{aligned}
&\int_k^{k+\frac{b}{2}}dx\int_l^{l+\sqrt{3}(x-k)}dyf\left(x,y\right)+\int_{k+\frac{b}{2}}^{k+b}dx\int_l^{l+\sqrt{3}(k+b-x)}dyf(x,y)\\
&=\int_k^{k+\frac{b}{2}}dx\left\{\left[\frac{\sqrt{3}}{2}bB_1\left(\frac{y-l}{\frac{\sqrt{3}}{2}b}\right)f(x,y)-\sum_{i=1}^{\lfloor M_y/2\rfloor}\frac{\left(\frac{\sqrt{3}}{2}b\right)^{2i}}{(2i)!}B_{2i}\left(\frac{y-l}{\frac{\sqrt{3}}{2}b}\right)\partial_y^{2i-1}f(x,y)\right]\Bigg|_l^{l+\sqrt{3}(x-k)}+R_{N_y}\right\}\\
&\quad+\int_{k+\frac{b}{2}}^{k+b}dx\left\{\left[\frac{\sqrt{3}}{2}bB_1\left(\frac{y-l}{\frac{\sqrt{3}}{2}b}\right)f(x,y)-\sum_{i=1}^{\lfloor M_y/2\rfloor}\frac{\left(\frac{\sqrt{3}}{2}b\right)^{2i}}{(2i)!}B_{2i}\left(\frac{y-l}{\frac{\sqrt{3}}{2}b}\right)\partial_y^{2i-1}f(x,y)\right]\Bigg|_l^{l+\sqrt{3}(k+b-x)}+R_{N_y}\right\}\\
&=\int_k^{k+\frac{b}{2}}dx\left[\frac{\sqrt{3}}{2}bB_1\left(\frac{x-k}{b/2}\right)f\left(x,l+\sqrt{3}(x-k)\right)-\sum_{i=1}^{\lfloor M_y/2\rfloor}\frac{\left(\frac{\sqrt{3}}{2}b\right)^{2i}}{(2i)!}B_{2i}\left(\frac{x-k}{b/2}\right)\partial_y^{2i-1}f\left(x,l+\sqrt{3}(x-k)\right)\right]\\
&\quad+\int_{k+\frac{b}{2}}^{k+b}dx\left[\frac{\sqrt{3}}{2}b\underbrace{B_1\left(\frac{k+b-x}{b/2}\right)}_{-B_1(\frac{x-k-b/2}{b/2})}f\left(x,l+\sqrt{3}(k+b-x)\right)\right.\\
&\qquad\left.-\sum_{i=1}^{\lfloor M_y/2\rfloor}\frac{\left(\frac{\sqrt{3}}{2}b\right)^{2i}}{(2i)!}\underbrace{B_{2i}\left(\frac{k+b-x}{b/2}\right)}_{B_{2i}(\frac{x-k-b/2}{b/2})}\partial_y^{2i-1}f\left(x,l+\sqrt{3}(k+b-x)\right)\right]\\
&\quad-\int_k^{k+b}dx\left[\frac{\sqrt{3}}{2}bB_1(0)f(x,l)-\sum_{i=1}^{\lfloor M_y/2\rfloor}\frac{\left(\frac{\sqrt{3}}{2}b\right)^{2i}}{(2i)!}B_{2i}(0)\partial_y^{2i-1}f(x,l)\right]+R_{N_y}\\
&=\frac{\sqrt{3}}{2}b\sum_{j=1}^{\lfloor M_x/2\rfloor}\frac{\left(\frac{b}{2}\right)^{2j-1}}{(2j)!}B_{2j}(\tfrac{x-k}{b/2})\left(\frac{d}{dx}\right)^{2j-2}f\left(x,l+\sqrt{3}(x-k)\right)\Bigg|_k^{k+\frac{b}{2}}\\
&\quad-\sum_{i=1}^{\lfloor M_y/2\rfloor}\sum_{j=1}^{\lfloor M_x/2\rfloor}\frac{\left(\frac{\sqrt{3}}{2}b\right)^{2i}\left(\frac{b}{2}\right)^{2j}}{(2i+2j)!}B_{2i+2j}(\tfrac{x-k}{b/2})\left(\frac{d}{dx}\right)^{2j-1}\partial_y^{2i-1}f\left(x,l+\sqrt{3}(x-k)\right)\Bigg|_k^{k+\frac{b}{2}}\\
&\quad-\frac{\sqrt{3}}{2}b\sum_{j=1}^{\lfloor M_x/2\rfloor}\frac{\left(\frac{b}{2}\right)^{2j-1}}{(2j)!}B_{2j}(\tfrac{x-k-b/2}{b/2})\left(\frac{d}{dx}\right)^{2j-2}f\left(x,l+\sqrt{3}(k+b-x)\right)\Bigg|_{k+\frac{b}{2}}^{k+b}\\
&\quad-\sum_{i=1}^{\lfloor M_y/2\rfloor}\sum_{j=1}^{\lfloor M_x/2\rfloor}\frac{\left(\frac{\sqrt{3}}{2}b\right)^{2i}\left(\frac{b}{2}\right)^{2j}}{(2i+2j)!}B_{2i+2j}(\tfrac{x-k-b/2}{b/2})\left(\frac{d}{dx}\right)^{2j-1}\partial_y^{2i-1}f\left(x,l+\sqrt{3}(k+b-x)\right)\Bigg|_{k+\frac{b}{2}}^{k+b}\\
&\quad+\frac{\sqrt{3}}{2}\frac{b}{2}\left[bB_1(\tfrac{x-k}{b})f(x,l)-\sum_{j=1}^{\lfloor M_x/2\rfloor}\frac{b^{2j}}{(2j)!}B_{2j}(\tfrac{x-k}{b})\partial_x^{2j-1}f(x,l)\right]\Bigg|_k^{k+b}\\
&\quad+\sum_{i=1}^{\lfloor M_y/2\rfloor}\frac{\left(\frac{\sqrt{3}}{2}b\right)^{2i}}{(2i)!}B_{2i}\left[bB_1(\tfrac{x-k}{b})\partial_y^{2i-1}f(x,l)-\sum_{j=1}^{\lfloor M_x/2\rfloor}\frac{b^{2j}}{(2j)!}B_{2j}(\tfrac{x-k}{b})\partial_y^{2i-1}\partial_x^{2j-1}f(x,l)\right]\Bigg|_k^{k+b}\\
&\quad+R_{M_x,M_y}.
\end{aligned}\tag{12}
$$

Unlike Eq. (11), the integrand in the second equal sign of Eq. (12) involves not only the arbitrary function $f(x,y)$ but also the Bernoulli polynomials, which leads to a more complicated result that does not respect the mirror symmetry and the rotational symmetry of equilateral triangle since we choose a specific orientation and ordering to expand the integral. To ensure the mirror symmetry, we consider the average of Eq. (12) and the result of its reflection about the angle bisector of the bottom left vertex of $S_\triangle$. To give a rotational symmetry-respecting formula, we consider the average of three integrals over the same minimal triangular period $S_\triangle$ written in three infinitesimal area elements,

$dxdy$, $d(-\frac{1}{2}x-\frac{\sqrt{3}}{2}y)d(\frac{1}{2}x-\frac{\sqrt{3}}{2}y)$, and $d(-\frac{1}{2}x+\frac{\sqrt{3}}{2}y)d(-x)$. Therefore, we focus on one vertex due to the symmetry consideration and use directional derivatives to give a coordinate-independent form of Eq. (12). Here we consider the bottom right vertex $(x,y)=(k+b,l)$ of $S_\triangle$ and define $\partial_\rightarrow$, $\partial_\uparrow$, $\partial_\searrow$, $\partial_\swarrow$ and $\partial_\searrow$ as the directional derivatives along directions whose angular deflections about the positive x axis are 0°, 90°, −60°, −150° and −30°, respectively, then we obtain

$$\begin{aligned}\int_{S_\triangle} dydx f(x,y) = \frac{1}{3}\frac{\sqrt{3}}{2}\frac{b^2}{2}\Bigg\{ & f + \sum_{i=1}^{\lfloor M/2\rfloor}\frac{b^{2i-1}}{(2i)!}B_{2i}\Big[-\partial_\rightarrow^{2i-1}f + (\frac{\sqrt{3}}{2}\partial_\uparrow)^{2i-1}f - \partial_\searrow^{2i-1}f + (\frac{\sqrt{3}}{2}\partial_\swarrow)^{2i-1}f\Big] \\ & -2\sum_{i=1}^{\lfloor M/2\rfloor}\sum_{j=1}^{\lfloor M/2\rfloor}\frac{b^{2i+2j-2}}{(2i)!\,(2j)!}B_{2i}B_{2j}\Big[\partial_\rightarrow^{2j-1}(\frac{\sqrt{3}}{2}\partial_\uparrow)^{2i-1}f + \partial_\searrow^{2j-1}(\frac{\sqrt{3}}{2}\partial_\swarrow)^{2i-1}f\Big] \\ & -\sum_{i=1}^{\lfloor M/2\rfloor}\sum_{j=1}^{\lfloor M/2\rfloor}\frac{b^{2i+2j-2}}{(2i+2j)!}B_{2i+2j}\Big[\partial_\searrow^{2j-1}(\frac{\sqrt{3}}{2}\partial_\searrow)^{2i-1}f + \partial_\rightarrow^{2j-1}(\frac{\sqrt{3}}{2}\partial_\searrow)^{2i-1}f \\ & \qquad + \partial_\searrow^{2j-1}(\frac{\sqrt{3}}{2}\partial_\uparrow)^{2i-1}f + \partial_\rightarrow^{2j-1}(\frac{\sqrt{3}}{2}\partial_\swarrow)^{2i-1}f\Big]\Bigg\} \\ & + R_z(\frac{2}{3}\pi) + R_z(\frac{4}{3}\pi) + R_M. \end{aligned} \tag{13}$$

The related terms at the other two vertices of $S_\triangle$ are given by either C_3 rotations with respect to out-of-plane direction or mirror reflections with respect to bisectors of the other two vertex angles, and we choose the same expansion order M in x and y to keep the rotational and mirror symmetries.

2. *Correction terms*

The 2D generalization of Euler-Maclaurin formula is similar to the 1D case, but the configurations of the boundaries of 2D regions differ from the 1D case. Take simple finite regions $T_{\square,\triangle}$ tiled by $S_{\square,\triangle}$ in Fig. 1a-d for example. We treat the integral of $f(x,y)$ over $T_{\square,\triangle}$ as the summation of that over all $S_{\square,\triangle}$, and the expansion of integral of $f(x,y)$ over $S_{\square,\triangle}$ can be viewed as $f(x,y)$ and its higher-order derivatives on the vertices of $S_{\square,\triangle}$ according to Eqs. (11) and (13). By collecting all the contributions from the vertices of all $S_{\square,\triangle}$, the integral of $f(x,y)$ over $T_{\square,\triangle}$ becomes $f(x,y)$ and its higher-order derivatives on the lattice points in Fig. 1e, f. Since there are several $S_{\square,\triangle}$ containing the same lattice point, the contribution from each lattice point can be categorized according to the configuration of its surroundings. For the rectangular tilings, we neglect the remainder term in Eq. (11) such that the contributions from those circled dots in Fig. 1e colored in black, yellow, red, and blue are given by

$$b_1 b_2 f, \tag{14}$$

$$\frac{1}{2}b_1 b_2 f - b_2\sum_{i=1}^{\lfloor M_x/2\rfloor}\frac{b_1^{2i}}{(2i)!}B_{2i}\partial_x^{2i-1}f, \tag{15}$$

$$\frac{1}{2}b_1 b_2 f - b_1\sum_{i=1}^{\lfloor M_y/2\rfloor}\frac{b_2^{2i}}{(2i)!}B_{2i}\partial_y^{2i-1}f, \tag{16}$$

$$\begin{aligned}&\frac{1}{4}b_1 b_2 f + \sum_{i=1}^{\lfloor M_x/2\rfloor}\sum_{j=1}^{\lfloor M_y/2\rfloor}\frac{b_1^{2i}b_2^{2j}}{(2i)!\,(2j)!}B_{2i}B_{2j}\partial_x^{2i-1}\partial_y^{2j-1}f \\ &-\frac{b_2}{2}\sum_{i=1}^{\lfloor M_x/2\rfloor}\frac{b_1^{2i}}{(2i)!}B_{2i}\partial_x^{2i-1}f - \frac{b_1}{2}\sum_{i=1}^{\lfloor M_y/2\rfloor}\frac{b_2^{2i}}{(2i)!}B_{2i}\partial_y^{2i-1}f,\end{aligned} \tag{17}$$

respectively. Note that all the other black dots in Fig. 1e have the same result as Eq. (14), while the contributions from other yellow, red and blue dots on the boundary $\partial T_{\square,\triangle}$ are given by the related mirror reflections of Eqs. (15), (16) and (17), respectively. Following the same procedure as the rectangular tiling, then the contributions from those circled dots in Fig. 1f colored in black, red and blue are given by

$$\frac{\sqrt{3}}{2}b^2 f, \tag{18}$$

$$\begin{aligned}&\frac{1}{2}\frac{\sqrt{3}}{2}b^2 f + \frac{1}{3}\frac{\sqrt{3}}{2}\frac{b^2}{2}\sum_{i=1}^{\lfloor M/2\rfloor}\frac{b^{2i-1}}{(2i)!}B_{2i} \\ &\times\Big[-2\partial_\rightarrow^{2i-1}f - 2\partial_\nearrow^{2i-1}f + 2(\frac{\sqrt{3}}{2}\partial_\swarrow)^{2i-1}f\Big],\end{aligned} \tag{19}$$

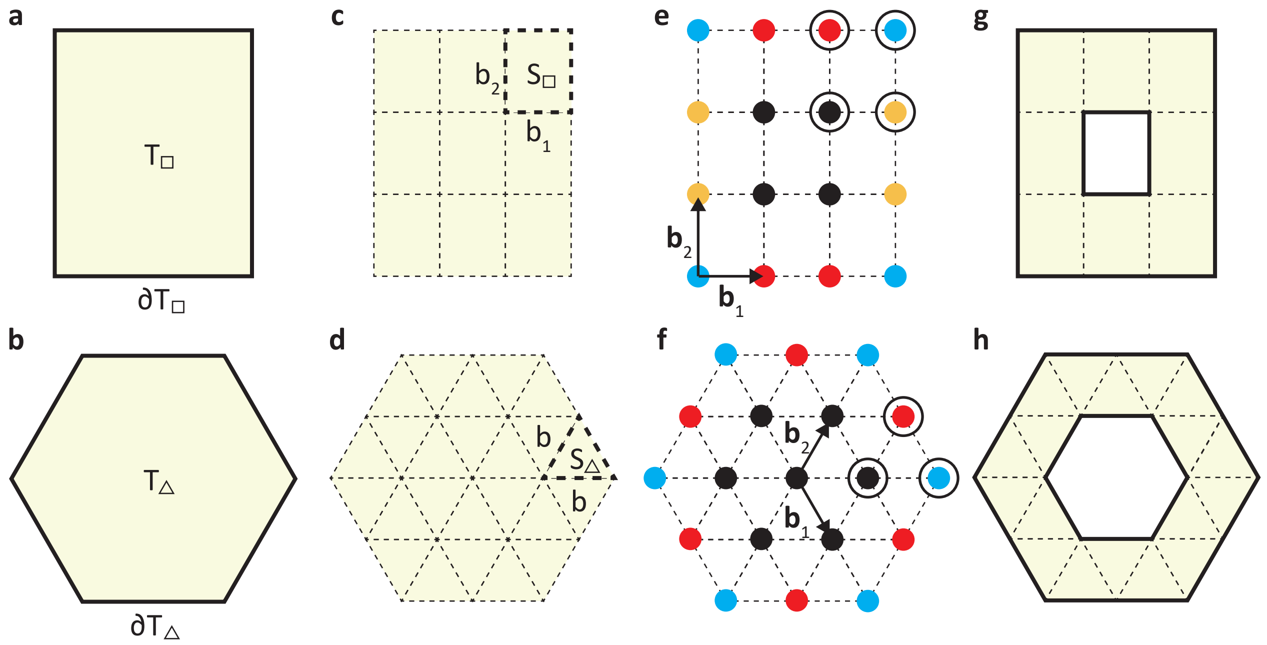

Supplementary Fig. 1. **2D generalization of Euler-Maclaurin formula.** **a**,**b**, Light yellow shaded regions $T_{\square,\triangle}$ whose boundary is denoted by $\partial T_{\square,\triangle}$. **c**,**d**, Tile $T_{\square,\triangle}$ by minimal dashed rectangles $S_\square$ with length of sides b_1 and b_2 and minimal dashed equilateral triangles $S_\triangle$ with length of side b, respectively. **e**,**f**, Black dots are the lattice points spanned by two lattice vectors $\mathbf{b}_1$ and $\mathbf{b}_2$ that are not on $\partial T_{\square,\triangle}$, and the other dots are those lattice points on $\partial T_{\square,\triangle}$. Points with different color means they have different contributions to the expansion of integral of arbitrary function $f(x,y)$ via Eqs. (11) and (13). **g**,**h**, Hollow regions $T_{\square,\triangle}$ as tilings of $S_{\square,\triangle}$ have outer and inner boundaries.

$$
\begin{aligned}
&\frac{1}{3}\frac{\sqrt{3}}{2}b^2\Bigg\{f+\frac{1}{2}\sum_{i=1}^{\lfloor M/2\rfloor}\frac{b^{2i-1}}{(2i)!}B_{2i}\Bigg[-2\partial_{\rightarrow}^{2i-1}f-\partial_{\searrow}^{2i-1}f\\
&-\partial_{\nearrow}^{2i-1}f+\Big(\frac{\sqrt{3}}{2}\partial_{\swarrow}\Big)^{2i-1}f+\Big(\frac{\sqrt{3}}{2}\partial_{\nwarrow}\Big)^{2i-1}f\Bigg]\\
&-\sum_{i=1}^{\lfloor M/2\rfloor}\sum_{j=1}^{\lfloor M/2\rfloor}\frac{b^{2i+2j-2}}{(2i)!\,(2j)!}B_{2i}B_{2j}\\
&\quad\times\Bigg[\partial_{\searrow}^{2j-1}\Big(\frac{\sqrt{3}}{2}\partial_{\swarrow}\Big)^{2i-1}f+\partial_{\nearrow}^{2j-1}\Big(\frac{\sqrt{3}}{2}\partial_{\nwarrow}\Big)^{2i-1}f\Bigg]\\
&-\frac{1}{2}\sum_{i=1}^{\lfloor M/2\rfloor}\sum_{j=1}^{\lfloor M/2\rfloor}\frac{b^{2i+2j-2}}{(2i+2j)!}B_{2i+2j}\\
&\quad\times\Bigg[\partial_{\searrow}^{2j-1}\Big(\frac{\sqrt{3}}{2}\partial_{\searrow}\Big)^{2i-1}f+\partial_{\searrow}^{2j-1}\Big(\frac{\sqrt{3}}{2}\partial_{\uparrow}\Big)^{2i-1}f\\
&\quad+\partial_{\nearrow}^{2j-1}\Big(\frac{\sqrt{3}}{2}\partial_{\nearrow}\Big)^{2i-1}f+\partial_{\nearrow}^{2j-1}\Big(\frac{\sqrt{3}}{2}\partial_{\downarrow}\Big)^{2i-1}f\Bigg]\Bigg\},
\end{aligned}
\tag{20}
$$

respectively. We use the directional derivatives again for simplicity, and $\partial_{\nearrow}$ and $\partial_{\nearrow}$ in Eqs. (19) and (20) are the directional derivatives along directions whose angular deflections about the positive x axis are 60° and 30°.

3. *Applications on infinite summations*

In order to simplify the infinite summation and integral over the whole 2D reciprocal space in Eq. (8), we now try to transform Eq. (8) as the boundary terms in the same manner as 1D Euler-Maclaurin formula. Notice that the expansion of integral of $f(x,y)$ over $T_{\square,\triangle}$ is linear in $f(x,y)$ in Eqs. (14) and (18) (except for the lattice points on the boundary of $T_{\square,\triangle}$), and its coefficient is determined by the area of unit cell (b_1b_2 and $\frac{\sqrt{3}}{2}b^2$), i.e., $|\mathbf{b}_1\times\mathbf{b}_2|$, which means the difference between the integral of $f(x,y)$ over $T_{\square,\triangle}$ divided by the area of unit cell $|\mathbf{b}_1\times\mathbf{b}_2|$ and the summation of $f(x,y)$ over all lattice points in $T_{\square,\triangle}$ only depends on $f(x,y)$ and its higher-order derivatives on the boundary $\partial T_{\square,\triangle}$. Therefore, we formally write our 2D generalization of Euler-Maclaurin formula for rectangular and triangular lattices in arbitrary $\mathbb{R}^2$ space as

$$
\begin{aligned}
&\sum_{\substack{\mathbf{r}=m_1\mathbf{b}_1+m_2\mathbf{b}_2\\ \mathbf{r}\in T_{\square,\triangle}}} f(\mathbf{r})-\int_{T_{\square,\triangle}}\frac{d^2r}{|\mathbf{b}_1\times\mathbf{b}_2|}f(\mathbf{r})\\
&=\text{correction terms on }\partial T_{\square,\triangle}\\
&\quad-\text{remainder terms.}
\end{aligned}
\tag{21}
$$

Notice that the finite region $T_{\square,\triangle}$ tiled by $S_{\square,\triangle}$ is not restricted to those shapes in Fig. 1a,b. In fact, Eq. (21) can be applied to arbitrary $T_{\square,\triangle}$ such as the hollow regions

in Fig. 1g, h since Eqs. (14) and (18) do not depend on the boundary, while different $T_{\square,\triangle}$ would result in the correction terms on $\partial T_{\square,\triangle}$ that differs from the above results. In general, we evaluate the correction terms on $\partial T_{\square,\triangle}$ by Eqs. (11) and (13), but we usually perform our 2D generalization of Euler-Maclaurin formula on the simple geometry like Fig. 1a, b, g, and h for the symmetry consideration.

Therefore, we calculate Eq. (8) in reciprocal space by connecting its right hand side to Eq. (21). We choose the arbitrary function $f(\mathbf{r})$ as the dyadic Green's function in reciprocal space $\overline{\overline{\mathbf{g}}}_0(\mathbf{G}+\mathbf{k}_\mathrm{B})$ divided by the area of unit cell in real space $|\mathbf{a}_1\times\mathbf{a}_2|$. Since the reciprocal lattice points are spanned by two reciprocal lattice vectors $\mathbf{b}_1 = 2\pi\frac{\mathbf{a}_2\times\mathbf{e}_z}{\mathbf{e}_z\cdot(\mathbf{a}_1\times\mathbf{a}_2)}$ and $\mathbf{b}_2 = 2\pi\frac{\mathbf{e}_z\times\mathbf{a}_1}{\mathbf{e}_z\cdot(\mathbf{a}_1\times\mathbf{a}_2)}$, the self-interaction reads

$$\overline{\overline{\mathbf{G}}}_0(\mathbf{0}) = \int\frac{d^2G}{(2\pi)^2}\overline{\overline{\mathbf{g}}}_0(\mathbf{G}) = \int\frac{|\mathbf{a}_1\times\mathbf{a}_2|}{(2\pi)^2}d^2G\frac{\overline{\overline{\mathbf{g}}}_0(\mathbf{G})}{|\mathbf{a}_1\times\mathbf{a}_2|} = \int\frac{d^2G}{|\mathbf{b}_1\times\mathbf{b}_2|}\underbrace{\frac{\overline{\overline{\mathbf{g}}}_0(\mathbf{G})}{|\mathbf{a}_1\times\mathbf{a}_2|}}_{f(\mathbf{G})}, \tag{22}$$

which means Eq. (8) is in the form of Eq. (21) such that we can utilize Eqs. (11) and (13) to evaluate Eq. (8) for square and triangular lattices by choosing an appropriate $T_{\square,\triangle}$. The absence of oscillatory term in $\overline{\overline{\mathbf{g}}}_0(\mathbf{G})$ results in the good convergence of Euler-Maclaurin formula even if we consider a small expansion order, and this is the reason we try to calculate the RDDI in the reciprocal space.

In fact, equation (21) can be applied in real space by adjusting the relationship among the lattice summation, integral and the correction terms in Eq. (21), but the existence of the oscillatory terms requires larger expansion order for the convergence. Taking the RDDI without the self-interaction such as Eq. (8) for example, the left-hand side of Eq. (8) is the summation on the whole lattice (without the origin) in real space, and it can be written in the following form by applying Eq. (21) on a 2D real lattice spanned by two direct lattice vectors $\mathbf{a}_1$ and $\mathbf{a}_2$

$$\begin{aligned}\sum_{\mathbf{R}\neq 0} & e^{-i\mathbf{k}_\mathrm{B}\cdot\mathbf{R}}\overline{\overline{\mathbf{G}}}_0(\mathbf{R}) \\ &= \left[\sum_{\substack{\mathbf{R}=m_1\mathbf{a}_1+m_2\mathbf{a}_2\\ \mathbf{R}\in T_{\square,\triangle}}} + \sum_{\substack{\mathbf{R}\notin T_{\square,\triangle}\\ \mathbf{R}\neq 0}}\right]e^{-i\mathbf{k}_\mathrm{B}\cdot\mathbf{R}}\overline{\overline{\mathbf{G}}}_0(\mathbf{R}) \\ &= \left[\int_{T_{\square,\triangle}}\frac{d^2R}{|\mathbf{a}_1\times\mathbf{a}_2|} + \sum_{\substack{\mathbf{R}\notin T_{\square,\triangle}\\ \mathbf{R}\neq 0}}\right]e^{-i\mathbf{k}_\mathrm{B}\cdot\mathbf{R}}\overline{\overline{\mathbf{G}}}_0(\mathbf{R}) \\ &\quad + \text{correction terms on } \partial T_{\square,\triangle} \\ &\quad - \text{remainder terms},\end{aligned} \tag{23}$$

where we need to choose a region $T_{\square,\triangle}$ that is periodically-tiled without the origin of real space (similar to Fig. 1g but its outer boundary extends infinitely) in avoid of the ill-defined unrenormalized self-interaction $\overline{\overline{\mathbf{G}}}_0(\mathbf{0})$. The last second line in Eq. (23) includes the integral of RDDI over $T_{\square,\triangle}$ and the lattice summation (without the origin) over a finite region, i.e., the complement of $T_\square$, and the former one requires model-dependent way to simplify the numerical computation. For instance, we separate the integral of RDDI over $T_{\square,\triangle}$ into the following two parts

$$\begin{aligned}\int_{T_{\square,\triangle}} & \frac{d^2R}{|\mathbf{a}_1\times\mathbf{a}_2|}e^{-i\mathbf{k}_\mathrm{B}\cdot\mathbf{R}}\overline{\overline{\mathbf{G}}}_0(\mathbf{R}) \\ &= \underbrace{\int\frac{d^2R}{|\mathbf{a}_1\times\mathbf{a}_2|}e^{-i\mathbf{k}_\mathrm{B}\cdot\mathbf{R}}\overline{\overline{\mathbf{G}}}_0(\mathbf{R})}_{\frac{1}{|\mathbf{a}_1\times\mathbf{a}_2|}\overline{\overline{\mathbf{g}}}_0(\mathbf{k}_\mathrm{B})} \\ &\quad - \int_{\mathbf{R}\notin T_{\square,\triangle}}\frac{d^2R}{|\mathbf{a}_1\times\mathbf{a}_2|}e^{-i\mathbf{k}_\mathrm{B}\cdot\mathbf{R}}\overline{\overline{\mathbf{G}}}_0(\mathbf{R}),\end{aligned} \tag{24}$$

where the last term has a singularity at the origin (self-interaction $\overline{\overline{\mathbf{G}}}_0(\mathbf{0})$) that needs to be treated individually for different electromagnetic environments. Also, the convergence of Euler-Maclaurin formula is determined by whether we can neglect the remainder terms in Eqs. (11) and (13) or not, and the existence of the oscillatory terms ($e^{-i\mathbf{k}_\mathrm{B}\cdot\mathbf{R}}\overline{\overline{\mathbf{G}}}_0(\mathbf{R})$) usually requires a larger expansion order M to achieve the desired precision. These issues imply that it is not that efficient to deal with Eq. (8) in real space.

4. *Collective Lamb shift at normal incidence*

As a demonstration of our method, we consider the collective Lamb shift of 2D square lattice in free space at normal incidence [6, 7]. The collective Lamb shift $\Delta_{\mathbf{k}_\mathrm{B}}$ and collective decay rate $\Gamma_{\mathbf{k}_\mathrm{B}}$ for a 2D square lattice are given by

$$\begin{aligned}\Delta_{\mathbf{k}_\mathrm{B}} & - \frac{i}{2}\Gamma_{\mathbf{k}_\mathrm{B}}\Big|_{\mathbf{k}_\mathrm{B}=0} \\ &= -\frac{3\pi\Gamma_0 c}{\omega_0 a^2}\mathbf{e}_d\cdot\left[\sum_{\mathbf{G}}\overline{\overline{\mathbf{g}}}_0(\mathbf{G}) - \frac{a^2}{(2\pi)^2}\int d^2G\,\overline{\overline{\mathbf{g}}}_0(\mathbf{G})\right]\cdot\mathbf{e}_d \\ &= -\frac{3\lambda^2\Gamma_0}{8\pi a^2}\left[\sum_{\mathbf{G}} - \frac{a^2}{(2\pi)^2}\int d^2G\right] \\ &\quad \times\left(1-\frac{\mathbf{G}^2}{2q^2}\right)\left[\frac{iq\Theta(q^2-\mathbf{G}^2)}{\sqrt{q^2-\mathbf{G}^2}} + \frac{q\Theta(\mathbf{G}^2-q^2)}{\sqrt{\mathbf{G}^2-q^2}}\right],\end{aligned} \tag{25}$$

where $\mathbf{e}_d$ is the unit vector of arbitrary in-plane polarization. Since the validity of 2D Euler-Maclaurin formula requires the smooth function and free-space dyadic Green's function in reciprocal space is ill-defined on the light cone, we choose a hollow region $T_\square$ that is periodically-tiled out of the light cone in which $\overline{\overline{\mathbf{g}}}_0(\mathbf{G}+\mathbf{k}_\mathrm{B})$ is smooth and

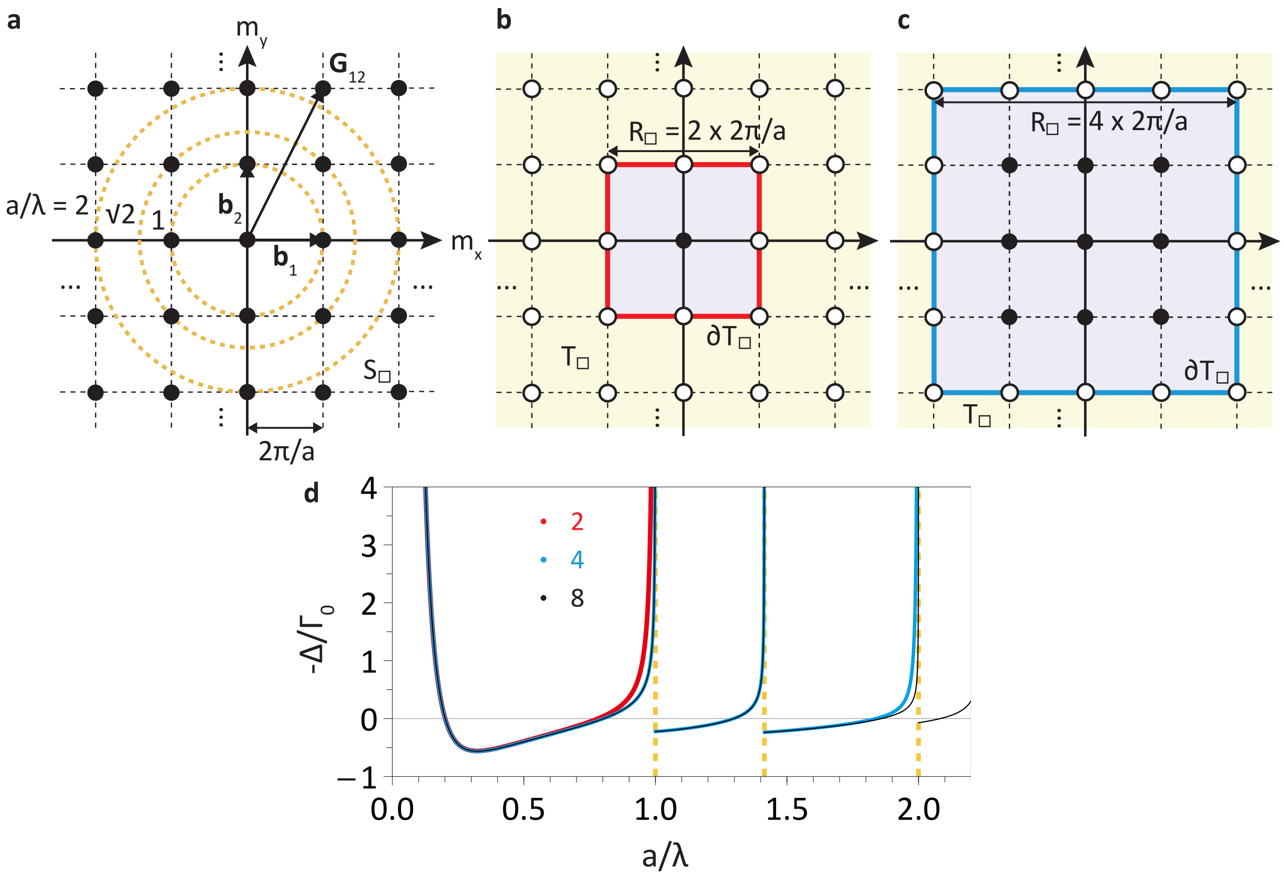

Supplementary Fig. 2. Demonstration of 2D generalization of Euler-Maclaurin formula for 2D free-space square lattice at normal incidence. **a**, 2D reciprocal space periodically tiled by the minimal period $S_\square$ (dashed square). Reciprocal lattice points $\mathbf{G}_{m_x m_y} = m_x\mathbf{b}_1 + m_y\mathbf{b}_2$ are spanned by two reciprocal lattice vectors $\mathbf{b}_1$ and $\mathbf{b}_2$ and marked as black dots. Yellow dashed circles indicate the smallest three light cones, defined by the ratio of atomic constant a to the resonant wavelength λ, intersecting the reciprocal lattice points. **b**,**c**, Light yellow shaded region is the tilings $T_\square$ whose outer boundary extends infinitely and inner boundary $\partial T_\square$ is shown as a red (blue) square centered at the origin with the length of sides $R_\square$ 2 (4) in unit of $2\pi/a$. Lattice points and the region of integration belong to $T_\square$ in the second line of Eq. (26) are marked as black circles and light yellow shaded region, and the rest are marked as black dots and light violet shaded region. **d**, Collective Lamb shift calculated within three different approximation boundaries $\partial T_\square$ whose length of sides $R_\square$ are 2 (red), 4 (blue) and 8 (black) in unit of $2\pi/a$, respectively. Red and blue curves associate with different choices of $T_\square$ in **b**,**c**, and three veritcal yellow dashed lines correspond to the smallest three light cones in **a**.

centered at the origin of reciprocal space (see Fig. 2b,c) to ensure the C_4 rotational symmetry of 2D square lattice to perform Eqs. (11) and (21) on the calculation of Eq. (25), then Eq. (25) can be divided into two parts by considering

$$\sum_{\mathbf{G}} - \frac{a^2}{(2\pi)^2}\int d^2G = \left[\sum_{\mathbf{G}\notin T_\square} - \int_{\mathbf{G}\notin T_\square} \frac{a^2 d^2G}{(2\pi)^2}\right] + \underbrace{\left[\sum_{\mathbf{G}\in T_\square} - \int_{T_\square} \frac{a^2 d^2G}{(2\pi)^2}\right]}_{=\text{ correction terms on } \partial T_\square}. \tag{26}$$

Similar to Eq. (23), the first term in Eq. (26) is the finite lattice summation and the integral over a finite region enclosed by the inner boundary of $T_\square$. Because the outer boundary of $T_\square$ extends infinitely in which the correction terms given by Eq. (21) vanish thanks to the ultraviolet frequency cutoff, we approximate the second term in Eq. (26) by the correction terms on the inner boundary $\partial T_\square$ and discard the negligible remainder term in Eq. (11), then we choose $\partial T_\square$ centered at the origin of reciprocal space (see Fig. 2b,c) with the same length of sides of $\partial T_\square$ in m_x and m_y directions, denoted by $R_\square$, to ensure the C_4 rotational symmetry of 2D square lattice.

Now we discuss how to decide a proper $\partial T_\square$ by comparing the prediction given by different lengths of sides $R_\square$, where we fix the expansion order M at 2 in both m_x and m_y to demonstrate the efficiency of our method. Note that the validity of the approximation given by Eq. (11) requires the small enough remainder term. Once the different $R_\square$ give the similar predictions within the desired error tolerance, we claim that our approximation is valid

since the difference between Eqs. (26) given by two different $R_\square$ and $R'_\square$ ($R_\square < R'_\square$) is

$$-\left[\sum_{\mathbf{G}\in T_\square/T'_\square} - \int_{T_\square/T'_\square} \frac{a^2 d^2 G}{(2\pi)^2}\right] + \begin{bmatrix}\text{correction terms} \\ \text{on } \partial T_\square\end{bmatrix} - \begin{bmatrix}\text{correction terms} \\ \text{on } \partial T'_\square\end{bmatrix}, \quad (27)$$

which is nothing but the difference between the exact expression and the related approximation, i.e., the remainder term, within the region $T_\square/T'_\square$ enclosed by the two approximation boundaries $\partial T_\square$ and $\partial T'_\square$.

In Fig. 2d, we find that different approximation regions $T_\square$ result in the same prediction of Eq. (25) except for the divergent behaviors. The reason why $R_\square = 2\times\frac{2\pi}{a}$ deviates from the correct divergent behavior at $\frac{a}{\lambda} = 1$ is that the divergence is well described by the dyadic Green's function at reciprocal lattice points while the approximation given by Eq. (11) with $R_\square = 2\times\frac{2\pi}{a}$ leaves only part of $\overline{\overline{\mathbf{g}}}_0(\mathbf{G})$ at reciprocal lattice points on $\partial T_\square$ in Fig. 2b. In addition, $R_\square = 2\times\frac{2\pi}{a}$ can only be used in subwavelength regime $\frac{a}{\lambda} < 1$ since the inner boundary of $T_\square$ needs to enclose the whole light cone whose diameter is $2\times\frac{2\pi}{\lambda}$ in order to make the remainder term negligible. Similarly, the calculation using $R_\square = 4\times\frac{2\pi}{a}$ coincides with the case of $R_\square = 8\times\frac{2\pi}{a}$ and starts to deviate from the correct result when $\frac{a}{\lambda} > 2$. In conclusion, we need to choose an appropriate $\partial T_\square$ whose interior contains the light cone and all potentially divergent terms determined by specific lattice constant and lattice geometry, and then we use larger $R_\square$ to check the convergence. In our experience, the calculation in the subwavelength regime using $R_\square = 4\times\frac{2\pi}{a}$ to $8\times\frac{2\pi}{a}$ is good enough (see Fig. 2d).

5. *Off-diagonal terms*

Although the RDDI between the same sublattice is present in the form of Eq. (26) in reciprocal space, it's not trivial to see whether we can still approximate the RDDI between different sublattices such as the off-diagonal terms in Eq. (10). We take honeycomb lattice with nearest separation a for example. The RDDI between two sublattices has the following form

$$\sum_{\mathbf{R}_2,\sqrt{3}a} e^{-i\mathbf{k}_\mathrm{B}\cdot(\mathbf{R}_2+\mathbf{b}_{21})}\overline{\overline{\mathbf{G}}}(\mathbf{R}_2+\mathbf{b}_{21}) = \frac{2}{\sqrt{3}}\frac{1}{(\sqrt{3}a)^2}\sum_{\mathbf{G},\frac{2}{\sqrt{3}}\frac{2\pi}{\sqrt{3}a}} e^{i\mathbf{G}\cdot\mathbf{b}_{21}}\overline{\overline{\mathbf{g}}}(\mathbf{G}+\mathbf{k}_\mathrm{B}), \quad (28)$$

where the subscripts of summation indicate the lengths of direct and reciprocal lattice vectors (see Fig. 3). Since the reciprocal lattice vectors $\mathbf{G} = m\mathbf{b}_1 + n\mathbf{b}_2$ result in three possible phase factors $e^{i\mathbf{G}\cdot\mathbf{b}_{21}} = e^{i\frac{2\pi}{3}(m+n)}$, Eq. (28)

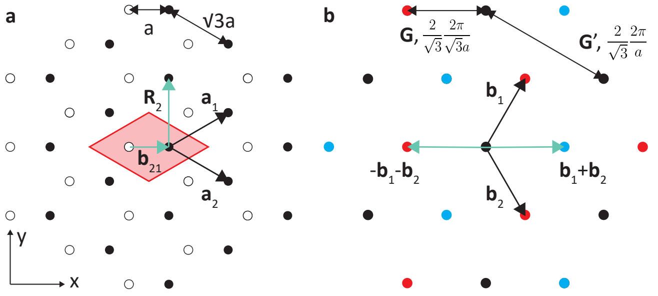

Supplementary Fig. 3. **Honeycomb lattice.** **a**, Honeycomb lattice with a nearest separation a in real space consists of two triangular sublattices 1 (white) and 2 (black) with the same lattice constant $\sqrt{3}a$. $\mathbf{R}_2$ means the direct lattice vectors of sublattice 2 and $\mathbf{b}_{21}$ is the nearest neighbor vector within the periodic unit cell (red shaded region). **b**, Reciprocal lattice vectors $\mathbf{G}$ of honeycomb lattices are spanned by $\mathbf{b}_1$ and $\mathbf{b}_2$ with the same length $\frac{2}{\sqrt{3}}\frac{2\pi}{\sqrt{3}a}$. Black, blue and red points form three triangular sublattices categorized by the global phase factors 1, $e^{i\frac{2\pi}{3}}$ and $e^{i\frac{4\pi}{3}}$ in Eq. (29), respectively.

can be decomposed into three parts

$$\sum_{\mathbf{G},\frac{2}{\sqrt{3}}\frac{2\pi}{\sqrt{3}a}} e^{i\mathbf{G}\cdot\mathbf{b}_{21}}\overline{\overline{\mathbf{g}}}(\mathbf{G}+\mathbf{k}_\mathrm{B}) = \sum_{\mathbf{G}',\frac{2}{\sqrt{3}}\frac{2\pi}{a}}\Big[\overline{\overline{\mathbf{g}}}(\mathbf{G}'+\mathbf{k}_\mathrm{B}) + e^{i\frac{2\pi}{3}}\overline{\overline{\mathbf{g}}}(\mathbf{G}'-\mathbf{b}_1-\mathbf{b}_2+\mathbf{k}_\mathrm{B}) + e^{i\frac{4\pi}{3}}\overline{\overline{\mathbf{g}}}(\mathbf{G}'+\mathbf{b}_1+\mathbf{b}_2+\mathbf{k}_\mathrm{B})\Big]. \quad (29)$$

Note that $1+e^{i\frac{2\pi}{3}}+e^{i\frac{4\pi}{3}} = 0$, which means we can assign the self-interactions for each dyadic Green's function in Eq. (29). That is, all infinite sums of RDDI in Eq. (10) can be approximated by our 2D generalization of Euler-Maclaurin formula, and the efficiency helps us explore the photonic band structures over the whole Brillouin zone.

SUPPLEMENTARY NOTE 3. NON-HERMITIAN BAND STRUCTURES

Recently, bulk Fermi arcs and paired exceptional points (EPs) have been theoretically proposed and experimentally realized in minimal two-band photonic crystals [8] by adding the radiation loss to generate EPs split from a Dirac point in the Hermitian regime, which arises from a C_{4v} symmetry-breaking perturbation, such as the change in lattice constant in one direction. However, we can not obtain EPs in the same way due to the inherent non-Hermiticity of RDDI in atomic arrays. Here we start from a non-Hermitian viewpoint to discuss the criteria for the emergence of EPs.

We consider a rectangular atomic lattice in free space. Each atom has a V-type energy level composed of one ground state $|g\rangle$ and two circularly-polarized excited states $|\pm\rangle = \mp(|x\rangle \pm i\,|y\rangle)/\sqrt{2}$ such that the system

supports two in-plane polarizations. In the circularly-polarized basis, the 2×2 effective Hamiltonian kernel is

$$\begin{aligned}\mathcal{H}_{\text{eff}}(\mathbf{k}_\text{B}) &= \hbar\left(\omega_0 - i\frac{\Gamma_0}{2}\right)\begin{pmatrix}1 & 0\\ 0 & 1\end{pmatrix} - \frac{3\pi\hbar\Gamma_0 c}{2\omega_0}\sum_{\mathbf{R}\neq 0} e^{-i\mathbf{k}_\text{B}\cdot\mathbf{R}} \\ &\times \begin{pmatrix} G_{0,xx}(\mathbf{R}) + G_{0,yy}(\mathbf{R}) + i\left[G_{0,xy}(\mathbf{R}) - G_{0,yx}(\mathbf{R})\right] & G_{0,yy}(\mathbf{R}) - G_{0,xx}(\mathbf{R}) + i\left[G_{0,xy}(\mathbf{R}) + G_{0,yx}(\mathbf{R})\right] \\ G_{0,yy}(\mathbf{R}) - G_{0,xx}(\mathbf{R}) - i\left[G_{0,xy}(\mathbf{R}) + G_{0,yx}(\mathbf{R})\right] & G_{0,xx}(\mathbf{R}) + G_{0,yy}(\mathbf{R}) - i\left[G_{0,xy}(\mathbf{R}) - G_{0,yx}(\mathbf{R})\right]\end{pmatrix} \\ &= \hbar(\omega_0 + \Omega_{\mathbf{k}_\text{B}})\begin{pmatrix}1 & 0\\ 0 & 1\end{pmatrix} + \hbar\Gamma_0\begin{pmatrix}0 & \kappa_{+-}(\mathbf{k}_\text{B})\\ \kappa_{-+}(\mathbf{k}_\text{B}) & 0\end{pmatrix}. \end{aligned} \tag{30}$$

Due to the dyadic form of Eq. (4), $G_{0,xy}(\mathbf{R}) = G_{0,yx}(\mathbf{R})$ leads to the identical diagonal terms for $|\pm\rangle$. In the absence of out-of-plane magnetic field $B\mathbf{e}_z$ that lifts the degeneracy in $|\pm\rangle$ by a Zeeman shift $\pm\hbar\mu B$, where μ is the magnetic moment, a real-valued $\hbar\mu B\sigma_z$ chiral term would not be induced in the effective Hamiltonian, and the emergence of degeneracy point only relies on the off-diagonal term in Eq. (30), as discussed in the main text.

To determine the criteria for the coalescence of two eigenstates of Eq. (30), we first use Eqs. (5) and (8) to express the non-Hermitian couplings $\kappa_{+-(-+)}(\mathbf{k}_\text{B})$ in reciprocal space as (assume the Bloch quasi-momentum $\mathbf{k}_\text{B}$ lies within the light cone)

$$\begin{aligned}\kappa_{+-(-+)}(\mathbf{k}_\text{B}) &= -\frac{3}{16\pi}\frac{\lambda^2}{|\mathbf{a}_1\times\mathbf{a}_2|}\left[\sum_{\mathbf{G}} - \int\frac{|\mathbf{a}_1\times\mathbf{a}_2|}{(2\pi)^2}d^2G\right] \\ &\times\left[\frac{i\Theta\left(q^2 - (\mathbf{G}+\mathbf{k}_\text{B})^2\right)}{\sqrt{1-\frac{1}{q^2}(\mathbf{G}+\mathbf{k}_\text{B})^2}} + \frac{\Theta\left((\mathbf{G}+\mathbf{k}_\text{B})^2 - q^2\right)}{\sqrt{\frac{1}{q^2}(\mathbf{G}+\mathbf{k}_\text{B})^2 - 1}}\right] \\ &\times\frac{1}{q^2}\left[(\mathbf{G}+\mathbf{k}_\text{B})_x \pm i(\mathbf{G}+\mathbf{k}_\text{B})_y\right]^2, \end{aligned} \tag{31}$$

which can be calculated by our 2D generalization of Euler-Maclaurin formula. The second line of Eq. (31) distinguishes between the contributions within and out of the light cone, which results in the anti-Hermitian and Hermitian couplings in Eq. (30) since only those modes lie within the light cone couple to outgoing photons in free space. For a square lattice, non-Hermitian couplings $\kappa_{+-(-+)}(\mathbf{k}_\text{B})$ vanish when $\mathbf{k}_\text{B}$ locates at high symmetry points Γ ($\mathbf{k}_\text{B} = 0$) in the first Brillouin zone due to the C_4 rotational symmetry, which means square lattices with in-plane polarizations have a non-Hermitian NDP at Γ and the corresponding eigenstates are distinct. This NDP is unstable since its existence relies on more conditions than that of EP in 2D system [9]. In our case, this NDP can be deformed into one or several EPs by breaking the C_4 rotational symmetry, which can be realized by deforming a square lattice into a rectangular or rhombic lattice.

According to the above discussion, the existence of NDP requires two things: one is the crystalline C_4 rotational symmetry, and the other is the absence of chiral term in the effective Hamiltonian. In fact, the latter is protected by the following symmetry

$$\sigma_x[\mathcal{H}_{\text{eff}}(\mathbf{k}_\text{B})]^T\sigma_x = \mathcal{H}_{\text{eff}}(\mathbf{k}_\text{B}), \tag{32}$$

and a 2×2 matrix A satisfying Eq. (32) is generally in the form of $A = a_0 I + a_x\sigma_x + a_y\sigma_y$, where $a_{0,x,y}$ can be complex. Combining Eq. (32) with the in-plane parity (inversion) symmetry $\mathcal{P}\mathcal{H}_{\text{eff}}(\mathbf{k}_\text{B})\mathcal{P}^{-1} = (-I)\mathcal{H}_{\text{eff}}(-\mathbf{k}_\text{B})(-I) = \mathcal{H}_{\text{eff}}(\mathbf{k}_\text{B})$ in two dimensions, which reverses the direction of polarization vectors and in-plane momentum, we obtain

$$\sigma_x[\mathcal{H}_{\text{eff}}(-\mathbf{k}_\text{B})]^T\sigma_x = \mathcal{H}_{\text{eff}}(\mathbf{k}_\text{B}), \tag{33}$$

whose left-hand side is the Hermitian-conjugated time-reversal operation $\mathcal{T}_+\mathcal{H}_{\text{eff}}(\mathbf{k}_\text{B})\mathcal{T}_+^{-1}$ with $\mathcal{T}_+\mathcal{T}_+^* = \sigma_x\sigma_x^* = 1$, and this equality means that $\mathcal{H}_{\text{eff}}(\mathbf{k}_\text{B})$ respects this Hermitian-conjugated time-reversal symmetry [10]. That is, both square and rectangular lattices respect this symmetry since it requires no higher crystalline symmetry, such that our system belongs to class $\text{AI}^\dagger$ [10]. As a result, NDP in a square lattice is protected by the C_4 rotational symmetry and the Hermitian-conjugated time-reversal symmetry, and NDP would be annihilated if one of these two symmetries is broken. Therefore, a Hermitian-conjugated time-reversal symmetry-breaking perturbation induced by an out-of-plane magnetic field can break the degeneracy at NDP, which is similar to the way an magnetic field opens a gap at the time-reversal symmetry-protected Dirac point in the Hermitian system.

SUPPLEMENTARY NOTE 4. ATOMIC LATTICE IN A RIBBON GEOMETRY

To calculate the OBC spectra in a ribbon geometry, we need to rewrite the RDDI in 1D reciprocal space (along the direction parallel to the open boundary). We integrate out the other two directions in Eq. (9) to obtain

$$\overline{\overline{\mathbf{G}}}_0(\mathbf{r}_\parallel,\mathbf{r}_\perp)=\int\frac{dk_\parallel}{(2\pi)^2}e^{i\mathbf{k}_\parallel\cdot\mathbf{r}_\parallel}\Bigg\{\left(\overline{\overline{\mathbf{I}}}-\frac{\mathbf{k}_\parallel^2}{q^2}\mathbf{e}_\parallel\otimes\mathbf{e}_\parallel\right)\left[\Theta(q^2-\mathbf{k}_\parallel^2)\frac{i\pi}{2}H_0^{(1)}(\sqrt{q^2-\mathbf{k}_\parallel^2}|\mathbf{r}_\perp|)+\Theta(\mathbf{k}_\parallel^2-q^2)K_0(\sqrt{\mathbf{k}_\parallel^2-q^2}|\mathbf{r}_\perp|)\right]$$
$$-\left(\mathbf{k}_\parallel\otimes\mathbf{r}_\perp+\mathbf{r}_\perp\otimes\mathbf{k}_\parallel\right)\frac{1}{|\mathbf{r}_\perp|}\frac{1}{q^2}\begin{bmatrix}-\Theta(q^2-\mathbf{k}_\parallel^2)\frac{\pi}{2}\sqrt{q^2-\mathbf{k}_\parallel^2}H_1^{(1)}(\sqrt{q^2-\mathbf{k}_\parallel^2}|\mathbf{r}_\perp|)\\+\Theta(\mathbf{k}_\parallel^2-q^2)i\sqrt{\mathbf{k}_\parallel^2-q^2}K_1(\sqrt{\mathbf{k}_\parallel^2-q^2}|\mathbf{r}_\perp|)\end{bmatrix}$$
$$-\mathbf{e}_\perp\otimes\mathbf{e}_\perp\begin{bmatrix}\Theta(q^2-\mathbf{k}_\parallel^2)\frac{i\pi}{4}(1-\frac{\mathbf{k}_\parallel^2}{q^2})\left(H_0^{(1)}(\sqrt{q^2-\mathbf{k}_\parallel^2}|\mathbf{r}_\perp|)-H_2^{(1)}(\sqrt{q^2-\mathbf{k}_\parallel^2}|\mathbf{r}_\perp|)\right)\\+\Theta(\mathbf{k}_\parallel^2-q^2)\frac{1}{2}(1-\frac{\mathbf{k}_\parallel^2}{q^2})\left(K_0(\sqrt{\mathbf{k}_\parallel^2-q^2}|\mathbf{r}_\perp|)+K_2(\sqrt{\mathbf{k}_\parallel^2-q^2}|\mathbf{r}_\perp|)\right)\end{bmatrix}\Bigg\}$$
$$=\int\frac{dk_\parallel}{2\pi}e^{i\mathbf{k}_\parallel\cdot\mathbf{r}_\parallel}\overline{\overline{\mathbf{g}}}_0(\mathbf{k}_\parallel,\mathbf{r}_\perp), \quad (34)$$

where $H_n^{(1)}$ is the Hankel function of the first kind, and K_n is the modified Bessel function of the second kind. Note that Eq. (34) is valid for the nonzero atomic separation $\mathbf{r}_\perp$ normal to the open boundary and used for calculating the off-diagonal blocks of effective Hamiltonian (see Eq. (36)). Owing to the exponentially decaying asymptotic behavior of the modified Bessel functions, the coupling between two 1D infinite atomic chains separated by $\mathbf{r}_\perp$ in a ribbon geometry (denoting their separation within the periodic unit cell, with a period $a_\parallel$, along the parallel direction as $\mathbf{b}_\parallel$),

$$\overline{\overline{\mathbf{t}}}(\mathbf{k}_\parallel,\mathbf{b}_\parallel,\mathbf{r}_\perp)$$
$$=-\frac{3\pi\hbar\Gamma_0 c}{\omega_0}\sum_{\mathbf{R}_\parallel}e^{-i\mathbf{k}_\parallel\cdot(\mathbf{R}_\parallel+\mathbf{b}_\parallel)}\overline{\overline{\mathbf{G}}}_0(\mathbf{R}_\parallel+\mathbf{b}_\parallel,\mathbf{r}_\perp)$$
$$=-\frac{3\pi\hbar\Gamma_0 c}{\omega_0}\sum_{\mathbf{G}_\parallel}\frac{1}{a_\parallel}e^{i\mathbf{G}_\parallel\cdot\mathbf{b}_\parallel}\overline{\overline{\mathbf{g}}}_0(\mathbf{G}_\parallel+\mathbf{k}_\parallel,\mathbf{r}_\perp), \quad (35)$$

can be easily addressed by summing over several reciprocal lattice vectors. For the self-interaction term $\overline{\overline{\mathbf{t}}}(\mathbf{k}_\parallel,\mathbf{b}_\parallel=0,\mathbf{r}_\perp=0)$, we should exclude $\mathbf{R}_\parallel=0$ term in the second line of Eq. (35) besides setting $\mathbf{r}_\perp,\mathbf{b}_\parallel=0$. We can determine whether the atomic lattice in a ribbon geometry has nonreciprocal coupling or not by Eq. (35). Since the reciprocity of RDDI results in $\overline{\overline{\mathbf{g}}}_0(\mathbf{k}_\parallel,\mathbf{r}_\perp)=\overline{\overline{\mathbf{g}}}_0{}^T(-\mathbf{k}_\parallel,-\mathbf{r}_\perp)$, this reciprocity leads to the reciprocal couplings in a ribbon geometry, $\overline{\overline{\mathbf{t}}}(\mathbf{k}_\parallel,\mathbf{b}_\parallel,\mathbf{r}_\perp)=\overline{\overline{\mathbf{t}}}^T(\mathbf{k}_\parallel,-\mathbf{b}_\parallel,-\mathbf{r}_\perp)$, only when $\mathbf{k}_\parallel=0$. Other reciprocal couplings comes from spatial symmetries in the real lattice space, such as the mirror symmetry.

1. *Energy spectra topology*

Following the same procedure as the 2D infinite atomic lattice, the effective non-Hermitian Hamiltonian of L 1D infinite atomic chains that supports a collective excitation with a fixed parallel momentum $\mathbf{k}_\parallel$ in a ribbon geometry is given by 1D Fourier transformation of Eq. (1)

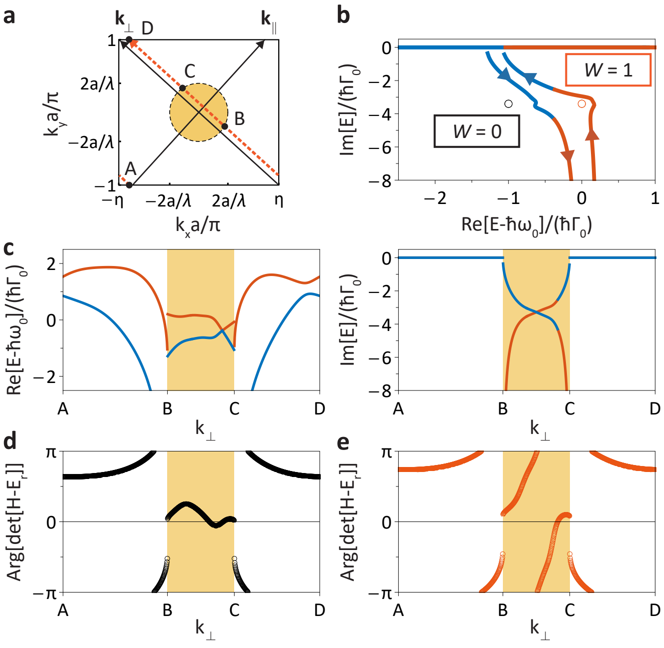

Supplementary Fig. 4. Bulk spectrum, band structure and winding numbers in a ribbon geometry. a,**b**, Momentum slice (orange dashed line, which forms a closed loop C on the T^2, 2D torus, Brillouin zone) and bulk spectrum of a 2D rectangular ($\eta=1.1$) lattice at fixed $\mathbf{k}_\parallel=0.1\pi/a$ (same as the top panel of FIG.2b in the main text). Black and orange circles indicate two topologically-distinct choices of the reference energy points E_r, and the corresponding spectral winding numbers are shown in Fig. 4d,e, respectively. **c**, Bulk eigenenergy as a function of $\mathbf{k}_\perp$, where the asymmetric behaviors at the opposite $\mathbf{k}_\perp$ reflect the nonreciprocity. Colors of two bands follow the same rule as those in **b**. **d**,**e**, Except for the discontinuities at the intersections of the light cone and the closed loop C, i.e., $\mathbf{k}_{\perp,\mathrm{B}}$ and $\mathbf{k}_{\perp,\mathrm{C}}$, the accumulated change in the arg[det[$\mathcal{H}(\mathbf{k}_\parallel,\mathbf{k}_\perp)-E_r$]] along the closed loop C represents the corresponding topological invariant $2\pi W(\mathbf{k}_\parallel,E_r)$.

$$\hat{\mathcal{H}}_L(\mathbf{k}_\parallel, \mathbf{r}^\perp) = \sum_{m=1}^{L} \sum_{\nu=x,y} \hbar\left(\omega_0 - i\frac{\Gamma_0}{2}\right) \hat{\sigma}^\dagger_{m\mathbf{k}_\parallel\nu} \hat{\sigma}_{m\mathbf{k}_\parallel\nu} + \sum_{m,n} \sum_{\mu,\nu=x,y} t_{\mu\nu}(\mathbf{k}_\parallel, \mathbf{b}^\parallel_{mn}, \mathbf{r}^\perp_{mn}) \hat{\sigma}^\dagger_{m\mathbf{k}_\parallel\mu} \hat{\sigma}_{n\mathbf{k}_\parallel\nu}, \tag{36}$$

whose kernel is a $2L \times 2L$ matrix. Numerical diagonalization of the Hamiltonian kernel of Eq. (36) $\mathcal{H}_L(\mathbf{k}_\parallel, \mathbf{r}_\perp)$ gives the OBC spectra $\sigma[\mathcal{H}_L(\mathbf{k}_\parallel, \mathbf{r}_\perp)]$ and OBC eigenstates $(\psi_x(\mathbf{r}_\perp), \psi_y(\mathbf{r}_\perp))^T$ of a single excited 2D atomic lattice in a ribbon geometry at a fixed $\mathbf{k}_\parallel$ in the main text.

The bulk spectrum of a 2D atomic lattice in a ribbon geometry at a fixed $\mathbf{k}_\parallel$ is obtained by considering its open boundary extends infinitely such that it becomes a 2D infinite lattice at a fixed $\mathbf{k}_\parallel$. Since the perpendicular direction also becomes infinite, its Hamiltonian can be characterized by a perpendicular momentum $\mathbf{k}_\perp$, and the bulk spectrum of this ribbon geometry is nothing but the 2D photonic band structures $\sigma[\mathcal{H}_{\text{eff}}(\mathbf{k}_\parallel, \mathbf{k}_\perp)]$ at a fixed $\mathbf{k}_\parallel$, e.g., a momentum slice denoted as the orange dashed line in Fig. 4a.

In Fig. 4, we discuss the details in the calculation of bulk band properties of a ribbon geometry, where we use the same parameter as Fig. 2b in the main text. Figure 4b presents the bulk energy spectrum parameterized by the perpendicular momentum $\mathbf{k}_\perp$, and Fig. 4c shows the $\mathbf{k}_\perp$ dependence of real and imaginary part of eigenenergy (band structures), where one of two bands diverges (see the denominators in Eqs. (5) and (36)) at the intersections of the light cone and the momentum slice, i.e., $\mathbf{k}_{\perp,\text{B}}$ and $\mathbf{k}_{\perp,\text{C}}$. These singularities are artifacts that arise from the infinite system, while it does not matter if we are interested in the near-resonant regime. Here we focus on the effect of RDDI and still keep these artifacts for the consistency of the theoretical model.

As shown in Fig. 4d,e, the discontinuities in the argument of $\det[\mathcal{H}_{\text{eff}}(\mathbf{k}_\parallel, \mathbf{k}_\perp) - E_r\mathbb{1}]$ at $\mathbf{k}_{\perp,\text{B}}$ and $\mathbf{k}_{\perp,\text{C}}$ make the determination of spectral winding number for a reference energy E_r ambiguous. Here we prove that these discontinuities at $\mathbf{k}_{\perp,\text{B}}$ and $\mathbf{k}_{\perp,\text{C}}$ cancel each other out. First notice that these discontinuities lead to a minus infinity to finite value jump in $\text{Re}[E_2(\mathbf{k}_\parallel, \mathbf{k}_{\perp,\text{B}})]$, a finite value to minus infinity jump in $\text{Re}[E_2(\mathbf{k}_\parallel, \mathbf{k}_{\perp,\text{C}})]$, a finite value to minus infinity jump in $\text{Im}[E_1(\mathbf{k}_\parallel, \mathbf{k}_{\perp,\text{B}})]$, and a minus infinity to finite value jump in $\text{Im}[E_1(\mathbf{k}_\parallel, \mathbf{k}_{\perp,\text{C}})]$ when $\mathbf{k}_\perp$ is swept from A to D in Fig. 4c. By rewriting $\det[\mathcal{H}_{\text{eff}}(\mathbf{k}_\parallel, \mathbf{k}_\perp) - E_r\mathbb{1}]$ as $(E_1(\mathbf{k}_\parallel, \mathbf{k}_\perp) - E_r)(E_2(\mathbf{k}_\parallel, \mathbf{k}_\perp) - E_r)$, the discontinuity in $\arg\det[\mathcal{H}_{\text{eff}}(\mathbf{k}_\parallel, \mathbf{k}_{\perp,\text{B}}) - E_r\mathbb{1}]$ is given by

$$\begin{aligned}
&\lim_{\mathbf{k}_\perp \to \mathbf{k}^+_{\perp,\text{B}}} \arg\det[\mathcal{H}_{\text{eff}}(\mathbf{k}_\parallel, \mathbf{k}_\perp) - E_r\mathbb{1}] - \lim_{\mathbf{k}_\perp \to \mathbf{k}^-_{\perp,\text{B}}} \arg\det[\mathcal{H}_{\text{eff}}(\mathbf{k}_\parallel, \mathbf{k}_\perp) - E_r\mathbb{1}] \\
&= \arg \lim_{\mathbf{k}_\perp \to \mathbf{k}^+_{\perp,\text{B}}} [E_1(\mathbf{k}_\parallel, \mathbf{k}_\perp) - E_r] - \arg \lim_{\mathbf{k}_\perp \to \mathbf{k}^-_{\perp,\text{B}}} [E_2(\mathbf{k}_\parallel, \mathbf{k}_\perp) - E_r] \\
&\quad + \left\{\arg \lim_{\mathbf{k}_\perp \to \mathbf{k}^+_{\perp,\text{B}}} [E_2(\mathbf{k}_\parallel, \mathbf{k}_\perp) - E_r] - \arg \lim_{\mathbf{k}_\perp \to \mathbf{k}^-_{\perp,\text{B}}} [E_1(\mathbf{k}_\parallel, \mathbf{k}_\perp) - E_r]\right\} \\
&= \arg \lim_{\mathbf{k}_\perp \to \mathbf{k}^+_{\perp,\text{B}}} i\text{Im}[E_1(\mathbf{k}_\parallel, \mathbf{k}_\perp)] - \arg \lim_{\mathbf{k}_\perp \to \mathbf{k}^-_{\perp,\text{B}}} \text{Re}[E_2(\mathbf{k}_\parallel, \mathbf{k}_\perp)] = \pi/2.
\end{aligned} \tag{37}$$

Since there is one continuous band, the first equality in Eq. (37) reduces to the discontinuity in the other band, where the divergent parts outside and within the light cone are a minus infinity in $\text{Re}[E_2(\mathbf{k}_\parallel, \mathbf{k}_{\perp,\text{B}})]$ and a minus infinity in $\text{Im}[E_1(\mathbf{k}_\parallel, \mathbf{k}_{\perp,\text{B}})]$, respectively. Similarly, the discontinuity in $\arg\det[\mathcal{H}_{\text{eff}}(\mathbf{k}_\parallel, \mathbf{k}_\perp) - E_r\mathbb{1}]$ is $-\pi/2$. These two discontinuities in $\arg\det[\mathcal{H}_{\text{eff}}(\mathbf{k}_\parallel, \mathbf{k}_{\perp,\text{C}}) - E_r\mathbb{1}]$ are consistent with the jumps in Fig. 4d,e at $\mathbf{k}_{\perp,\text{B}}$ and $\mathbf{k}_{\perp,\text{C}}$ such that these discontinuities cancel each other out exactly, which makes the spectral winding number integer-valued. Therefore, we can easily determine the respective spectral winding numbers in Fig. 4d,e as $W(\mathbf{k}_\parallel, E_r) = 0$ and $W(\mathbf{k}_\parallel, E_r) = 1$ by the overall change in the $\arg[\det[\mathcal{H}_{\text{eff}}(\mathbf{k}_\parallel, \mathbf{k}_\perp) - E_r]]$ along the closed loop C. We note that these discontinuities at the light cone cancel each other out no matter what lattice configurations we considered.

2. *Ribbon geometry with a finite width*

Besides the numerical diagonalization, OBC eigenstates can be solved by the following recursive sequence [11, 12] (here we review the previous method in terms of our system)

$$\begin{aligned}
E_{\text{OBC}}\psi_{m\mu} &= \hbar\left(\omega_0 - i\frac{\Gamma_0}{2}\right)\psi_{m\mu} + \sum_{\nu=x,y} t_{\mu\nu}(\mathbf{k}_\parallel, 0, 0)\psi_{m,\nu} \\
&\quad + \sum_{j=1}^{L-1} \sum_{\nu=x,y} t_{\mu\nu}(\mathbf{k}_\parallel, \mathbf{b}^\parallel_{m,m+j}, -j\boldsymbol{a}_\perp)\psi_{m+j,\nu} \\
&\quad + \sum_{j=1}^{L-1} \sum_{\nu=x,y} t_{\mu\nu}(\mathbf{k}_\parallel, \mathbf{b}^\parallel_{m,m-j}, j\boldsymbol{a}_\perp)\psi_{m-j,\nu},
\end{aligned} \tag{38}$$

where $\psi_{m\mu} = \psi_\mu(m\boldsymbol{a}_\perp)$ for $1 \le m \le L,\ \mu = x, y$ and $\boldsymbol{a}_\perp$ is the lattice vector normal to the open boundary. The ansatz to Eq. (38) is

$$\psi_{m\mu} = \beta^m A_\mu(\beta),\ (\mu = x, y) \tag{39}$$

By inserting Eq. (39) into Eq. (38), nontrivial solutions of the coefficients $A_\mu(\beta)$ correspond to the following characteristic equation

$$\begin{aligned}\mathcal{H}_{\mu\nu}(\beta) = {}& \hbar\left(\omega_0 - i\frac{\Gamma_0}{2}\right)\delta_{\mu\nu} + \sum_{\nu=x,y} t_{\mu\nu}(\mathbf{k}_\parallel, 0, 0)\\ &+ \sum_{j=1}^{L-1} t_{\mu\nu}(\mathbf{k}_\parallel, \mathbf{b}^\parallel_{0j}, -j\boldsymbol{a}_\perp)\beta^j\\ &+ \sum_{j=1}^{L-1} t_{\mu\nu}(\mathbf{k}_\parallel, \mathbf{b}^\parallel_{j0}, j\boldsymbol{a}_\perp)\beta^{-j},\end{aligned} \tag{40}$$

$$\det[\mathcal{H}(\beta) - E_{\rm OBC}] = 0, \tag{41}$$

$$\sum_{\nu=x,y} \mathcal{H}_{\mu\nu}(\beta)A_\nu(\beta) = E_{\rm OBC}A_\mu(\beta), \tag{42}$$

where $\mathcal{H}(\beta)$ is a 2×2 matrix (two in-plane polarizations). Since there are $2 \times [(L-1) + (L-1)] = 4(L-1)$ (two in-plane polarizations $\times$ (leftward interaction range + rightward interaction range)) solutions of β to Eq. (41), OBC eigenstate is composed of the linear combination of these $4(L-1)$ modes

$$\psi^{\rm OBC}_{m\mu} = \sum_{i=1}^{4(L-1)} (\beta_i)^m A_{i\mu}, \tag{43}$$

where β_i are sorted by their moduli in the ascending order and $A_{i\mu} = A_\mu(\beta_i)$, and the OBCs are $\psi^{\rm OBC}_{m\mu} = 0$ for $-L+2 \le m \le 0$, $L+1 \le m \le 2L-1$, and $\mu = x, y$. According to these OBCs, the existence of nontrivial solutions of $A_{i\mu}$ requires

$$\begin{vmatrix} (\beta_1)^{-L+2} & \cdots & (\beta_{4(L-1)})^{-L+2} \\ (\beta_1)^{-L+2}\frac{E_{\rm OBC}-\mathcal{H}_{xx}(\beta_1)}{\mathcal{H}_{xy}(\beta_1)} & \cdots & (\beta_{4(L-1)})^{-L+2}\frac{E_{\rm OBC}-\mathcal{H}_{xx}(\beta_{4(L-1)})}{\mathcal{H}_{xy}(\beta_{4(L-1)})} \\ \vdots & \vdots & \vdots \\ (\beta_1)^0 & \cdots & (\beta_{4(L-1)})^0 \\ (\beta_1)^0\frac{E_{\rm OBC}-\mathcal{H}_{xx}(\beta_1)}{\mathcal{H}_{xy}(\beta_1)} & \cdots & (\beta_{4(L-1)})^0\frac{E_{\rm OBC}-\mathcal{H}_{xx}(\beta_{4(L-1)})}{\mathcal{H}_{xy}(\beta_{4(L-1)})} \\ (\beta_1)^{L+1} & \cdots & (\beta_{4(L-1)})^{L+1} \\ (\beta_1)^{L+1}\frac{E_{\rm OBC}-\mathcal{H}_{xx}(\beta_1)}{\mathcal{H}_{xy}(\beta_1)} & \cdots & (\beta_{4(L-1)})^{L+1}\frac{E_{\rm OBC}-\mathcal{H}_{xx}(\beta_{4(L-1)})}{\mathcal{H}_{xy}(\beta_{4(L-1)})} \\ \vdots & \vdots & \vdots \\ (\beta_1)^{2L-1} & \cdots & (\beta_{4(L-1)})^{2L-1} \\ (\beta_1)^{2L-1}\frac{E_{\rm OBC}-\mathcal{H}_{xx}(\beta_1)}{\mathcal{H}_{xy}(\beta_1)} & \cdots & (\beta_{4(L-1)})^{2L-1}\frac{E_{\rm OBC}-\mathcal{H}_{xx}(\beta_{4(L-1)})}{\mathcal{H}_{xy}(\beta_{4(L-1)})} \end{vmatrix} = 0. \tag{44}$$

Note that β_i, $E_{\rm OBC}$ and $A_{i\mu}$ are self-consistently determined by Eqs. (41) to (44). Rewriting β as $e^{i(\mathbf{k}_\perp + i\boldsymbol{\kappa}_\perp)\cdot\boldsymbol{a}_\perp}$, the modes with $|\beta| = 1$ ($|\beta| \neq 1$) in the thermodynamic limit are extended Bloch modes (non-Bloch modes), and $\mathcal{H}(\beta)$ becomes the non-Bloch real-space Hamiltonian.

To see how the long-range interactions modify the non-Bloch band theory, here we briefly review the celebrated generalized Brillouin zone [11, 12, 17] in the systems with one degree of freedom in the unit cell and finite-range (μ) couplings. The leading term in Eq. (44) now becomes $\beta_1^{-\mu+1}\beta_2^{-\mu+2}\cdots\beta_{2\mu-1}^{L+\mu-1}\beta_{2\mu}^{L+\mu}$. If each power function of β_i in $\beta_1^{-\mu+1}\beta_2^{-\mu+2}\cdots\beta_{2\mu-1}^{L+\mu-1}\beta_{2\mu}^{L+\mu}$ is exponentially growing in L, i.e., β_i has a fixed length scale, the term closest to $\beta_1^{-\mu+1}\beta_2^{-\mu+2}\cdots\beta_{2\mu-1}^{L+\mu-1}\beta_{2\mu}^{L+\mu}$ would be its interchange of two adjacent β_μ and $\beta_{\mu+1}$ whose magnitudes are closest to unity. To make Eq. (44) vanish in the thermodynamic limit, these two leading terms should cancel each other out, which results in the condition $|\beta_\mu| = |\beta_{\mu+1}|$. The trajectories of β_μ and $\beta_{\mu+1}$, parameterized by their phases and known as generalized Brillouin zone, form the continuum bands $\sigma[\mathcal{H}(\beta)]$ in the thermodynamic limit. The conditions for the continuum bands depend on the symmetry class of the non-Hermitian matrices. For instance, the non-Bloch modes β and β^{-1} appear in pair in the presence of reciprocal couplings, where the non-Hermitian Hamiltonians belong to class $\mathrm{AI}^\dagger$ or $\mathrm{AII}^\dagger$ [10], and the conditions for the continuum bands would be modified [17].

However, the size independence of the decay length of β breaks down in the presence of long-range interactions. The reason that this size independence of β is reasonable in the standard non-Bloch band theory is the size independence of both the characteristic equation (Eq. (41)) and the numerically calculated OBC spectra. Once we have the size-independent characteristic equation and the size-dependent OBC spectra, Eq. (41) predicts the presence of β with size-dependent decay

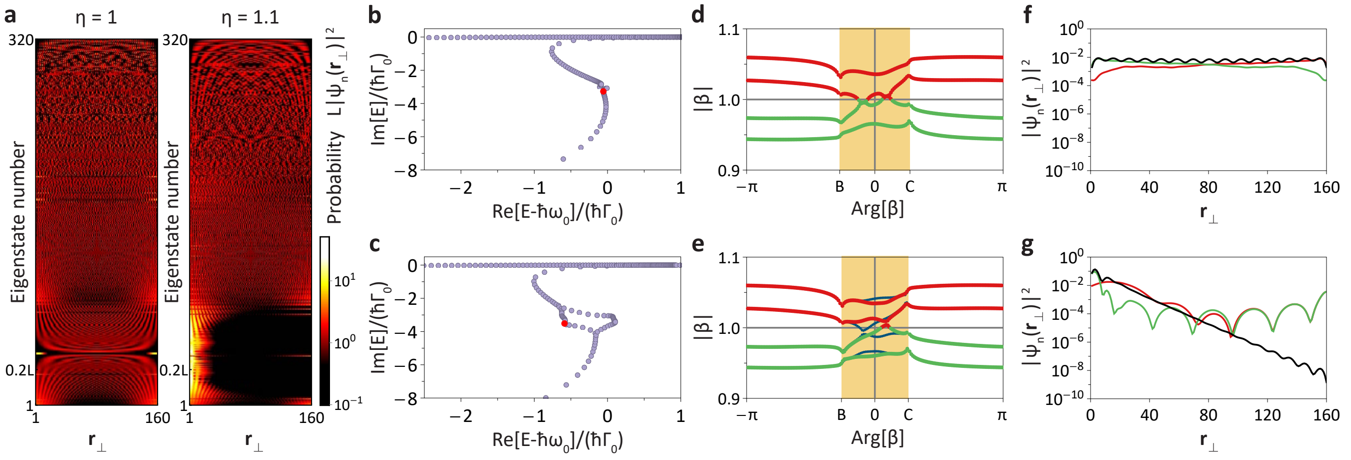

Supplementary Fig. 5. Open boundary eigenstates and β. Open boundary eigenstates (**a**) and spectra (**b**,**c**) of 2D square and rectangular atomic lattices in ribbon geometries at 160 unit cells. The plots are obtained with the same parameters as Fig. 3 in the main text. In the left (right) panel of **a**, the modes with complex eigenenergies are delocalized (localized) in 2D square (rectangular) atomic lattices, where the eigenenergy of the $(0.2L)$th eigenstate is colored in red in **b** (**c**). **d**-**g**, The $4(L-1)$ solutions of β (**d**,**e**) and OBC eigenstate profiles (**f**,**g**) of the $(0.2L)$th eigenstates of 2D square (**d**,**f**) and rectangular (**e**,**g**) atomic lattices in ribbon geometries. The modes with $|\beta|>1$ ($|\beta|<1$) that are localized at the boundary in increasing (decreasing) $\mathbf{r}_\perp$ direction are colored in red (green). Owing to the mirror symmetry, those β and β^{-1} (blue) in **d** are paired, while β in **e** are not paired in the presence of non-reciprocal coupling. In contrast to the tight-binding model, OBC eigenstates (black) in **f**,**g** are composed of several β in the presence of long-range interaction, where the contributions from the modes with $|\beta|>1$ and $|\beta|<1$ are colored in red and green, respectively.

length, where the competition between the β with constant and size-dependent decay lengths results in the critical non-Hermitian skin effect [14, 15]. In our case, both the characteristic equation and OBC spectra are size-dependent, which leads to β with no fixed scales, and the number of β is also size-dependent. As a result, each OBC eigenstate is not dominated by two but several β, which means the decaying behaviors of β do not directly reflect the decaying behaviors of OBC eigenstates due to the interference among several β. We note that the size dependence of OBC spectra and β should be self-consistently determined by Eqs. (41) to (44), while we can first numerically diagonalize the Hamiltonian kernel of Eq. (36) $\mathcal{H}_L(\mathbf{k}_\parallel,\mathbf{r}_\perp)$ to see the size dependence of OBC spectra such that we can qualitatively determined the size dependence of β.

Here we choose the $(0.2L)$th eigenstate of 2D square and rectangular lattices in a ribbon geometry open on the $(\bar{1}1)$ plane (see Fig. 5a-c) to numerically demonstrate the above discussions. In Fig. 5d-g, we numerically calculate the $4(L-1)$ solutions of β for square and rectangular lattices in ribbon geometries at $L=160$ unit cells. Due to the mirror symmetry over the open boundary of a square lattice in a ribbon geometry, the effective coupling is reciprocal and the Hamiltonian kernel of Eq. (36) $\mathcal{H}_L(\mathbf{k}_\parallel,\mathbf{r}_\perp)$ respects the Hermitian-conjugated time-reversal symmetry [10]

$$\mathcal{T}_+[\mathcal{H}_L(\mathbf{k}_\parallel,\mathbf{r}_\perp)]^T\mathcal{T}_+^{-1}=\mathcal{H}_L(\mathbf{k}_\parallel,\mathbf{r}_\perp), \tag{45}$$
$$\mathcal{T}_+\mathcal{T}_+^*=+1, \tag{46}$$

where a unitary matrix $\mathcal{T}_+=\mathbb{1}\otimes\sigma_z$ when $\mathcal{H}_L(\mathbf{k}_\parallel,\mathbf{r}_\perp)$ is defined in the linearly-polarized basis along the parallel and perpendicular directions. The left-hand side of Eq. (45) is the Hamiltonian kernel after the mirror reflection with respect to the open boundary in a ribbon geometry, which reverses the lattice site $\mathbf{r}_\perp$, leaves the parallel polarization unchanged, and flips the perpendicular polarization. That is, a non-Hermitian Hamiltonian kernel $\mathcal{H}_L(\mathbf{k}_\parallel,\mathbf{r}_\perp)$ of a square lattice in a ribbon geometry that respects the mirror symmetry over the open boundary belongs to class AI† [10]. Since each β becomes β^{-1} when the lattice sites $\mathbf{r}_\perp$ are reversed, the corresponding Hamiltonian $\mathcal{H}(\beta)$ respects

$$\sigma_z[\mathcal{H}(\beta)]^T\sigma_z=\mathcal{H}(\beta^{-1}). \tag{47}$$

Accordingly, when β is a solution to the characteristic equation Eq. (41), we have

$$\begin{aligned}\det[\mathcal{H}(\beta^{-1})-E_{\mathrm{OBC}}]&=\det[\sigma_z[\mathcal{H}(\beta)]^T\sigma_z-E_{\mathrm{OBC}}]\\&=\det[\mathcal{H}(\beta)-E_{\mathrm{OBC}}]\\&=0,\end{aligned} \tag{48}$$

which means β^{-1} is also a solution to Eq. (41). Therefore, the degenerate β and β^{-1} appear in pair in the presence of reciprocal couplings [17], which is also shown in Fig. 5d, where the numerically calculated $4(L-1)$ solutions of β coincide with their reciprocal β^{-1} colored in blue (right behind those red and green dots). By contrast, $4(L-1)$ soultions of β are not paired in Fig. 5e since the effective coupling in a rectangular lattice in a ribbon geometry is nonreciprocal.

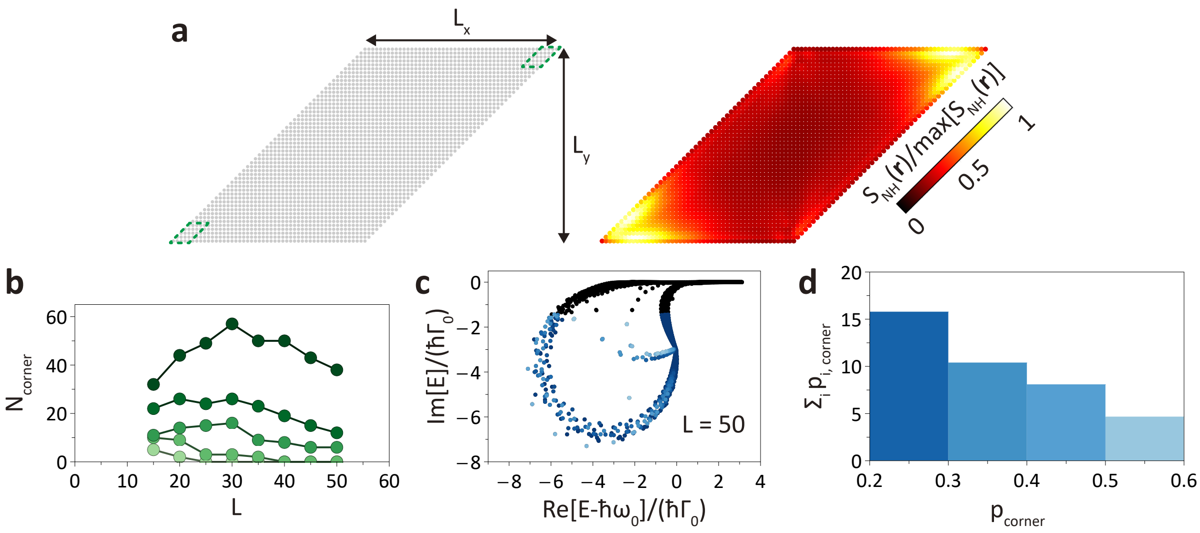

Supplementary Fig. 6. **Corner accumulation in a square lattice with parallelogram-shaped boundary.** **a**, Schematic of a 2D square lattice (left) with parallelogram-shaped boundary at system size $L_xL_y = L^2$ ($L_x = L_y = L$), where the green dashed parallelograms indicate the corner we considered. At $L = 50$, the average spatial distribution $S_{\rm NH}(\mathbf{r})$ (right) shows the accumulation at the corner with an acute angle. **b**, Scaling behavior of $N_{\rm corner}$. The lighter to the darker color gradients represent that a corner mode is identified when its population within the corner is larger than 0.5, 0.4, 0.3, 0.2, and 0.1, respectively. **c**, OBC spectrum at $L = 50$. The $N' = [\pi(a/\lambda)^2/\eta] \times 2L^2$ modes used for calculating $S_{\rm NH}(\mathbf{r})$ are shown in blue, and the other modes are in black. **d**, Contributions to the corner accumulation from the modes with $p_{i,\rm corner} \in [0.1j, 0.1(j+1)]$ ($2 \le j \le 4$, dark) and $p_{\rm corner} \ge 0.5$ (light). The modes in **c** are colored in the same manner as those in **d**, where the darkest blue corresponds to $p_{i,\rm corner} < 0.2$.

We can also reconstruct each OBC eigenstate by these β. In Fig. 5h, it is clear to see that the decaying behavior of OBC eigenstate (skin mode) does not arise from those decaying modes with $|\beta| < 1$ (green) but from the interference of the growing and decaying β. Also, delocalized OBC eigenstate in Fig. 5g is not composed of two degenerate β but several β. Therefore, we conclude that the OBC eigenstates of a non-Hermitian Hamiltonian in the presence of long-range interactions can not be described by two dominant β, which implies that the generalized Brillouin zone is inapplicable when the coupling range is not fixed.

SUPPLEMENTARY NOTE 5. FINITE-SIZE TWO-DIMENSIONAL ATOMIC LATTICES

1. *Effect of long-range interaction*

Here we explore the accumulation appearing at the corner with an acute angle in Fig. 4c in the main text. First, we will show that the accumulation does not exhibit the non-Hermitian skin effect. Next, we will reveal that the accumulation is due to the effect of the long-range interaction.

We start by choosing the bottom left 5^2 and the top right 5^2 sites together as the corner, indicated by the green dashed parallelograms in Fig. 6a (left). An eigenmode is identified as a corner mode when its population within the corner exceeds a threshold. By this criterion, we select the corner modes among $N' = [\pi(a/\lambda)^2/\eta] \times 2L^2$ modes which we use to calculate the average probability distribution, $S_{\rm NH}(\mathbf{r})$, in Fig. 6a (right). As a result, the number of corner modes $N_{\rm corner}$ as a function of L is shown in Fig. 6b. It is evident that these eigenstates at the corner exhibit neither the higher-order non-Hermitian skin effect with $\mathcal{O}(L^2)$ skin corner modes [16] nor the second-order non-Hermitian skin effect with $\mathcal{O}(L)$ corner modes [17]. This result is independent of the threshold.

To understand the origin of the corner accumulation, we have to know which modes make major contributions to the corner accumulation. The contributions depend on an i-th mode's population within the corner (denoted as $p_{i,\rm corner}$) and the number of such a mode. In Fig. 6c and 6d, we define the bottom left 10^2 and the top right 10^2 sites together as the corner. We first note that the modes with $p_{i,\rm corner} < 0.2$ would not lead to the corner accumulation since these modes are quite uniformly distributed. For the modes with $p_{i,\rm corner} \ge 0.5$, they have large population within the corner but are few in number. Therefore, their contribution to the corner accumulation $\sum_{p_{i,\rm corner}\ge 0.5} p_{i,\rm corner}$ is much less dominant in comparsion with the modes with $p_{i,\rm corner} \in [0.2, 0.5]$ (Fig. 6d). Therefore, the modes with $0.2 \le p_{i,\rm corner} \le 0.5$ contribute substantially to the corner accumulation. According to Fig. 6c, we can find that these modes correspond to the bulk modes in the vicinity of the light cone. Since the light cone arises from the long-range interaction, the long-range interaction should be responsible to this slight

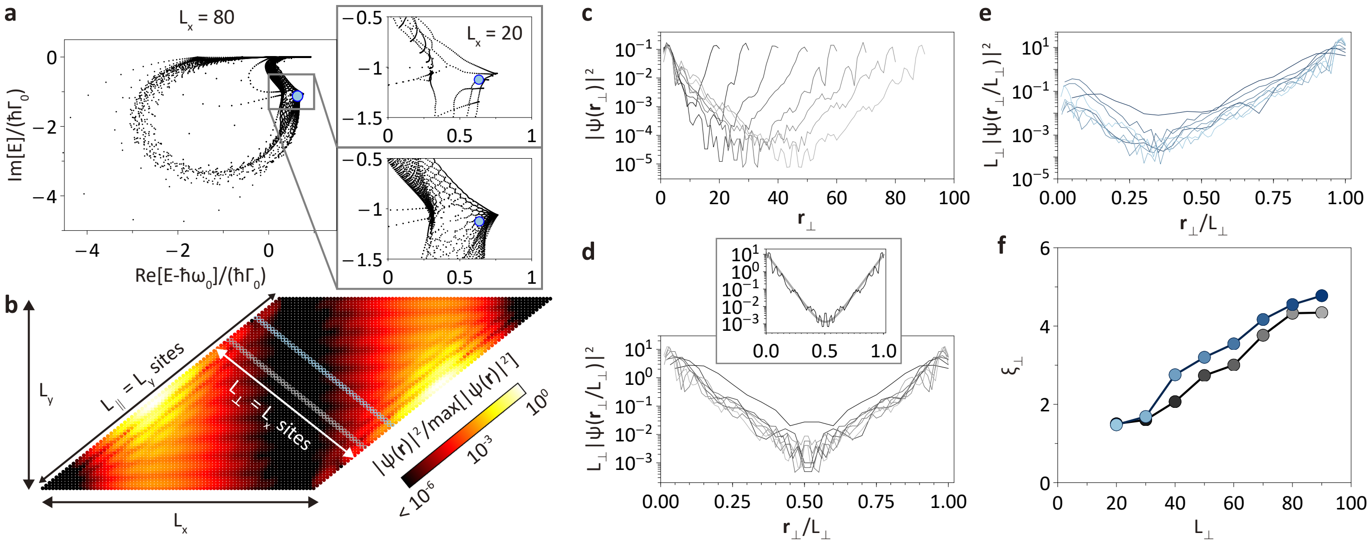

Supplementary Fig. 7. **Scaling behavior of the skin mode in a rectangular lattice with parallelogram-shaped boundary. a**, Spectrum of a rectangular lattice ($a/\lambda = 0.3$, $\eta = 0.8$) with system size $L_x \times L_y = 80 \times 70$. The upper (lower) inset is a zoomed-in figure of the chosen skin mode, denoted as the blue dot, at $L_x = 20$ ($L_x = 80$). **b**, Probability distribution of the chosen skin mode for $L_x = 80$ and $L_y = 70$. Two different 1D atomic chains are labeled by gray and blue circles, respectively. The sites along the 1D chain are labeled by $\mathbf{r}_\perp$. **c**,**d**, Normalized probability distribution $|\psi(\mathbf{r}_\perp)|^2$ (**c**) and the rescaled probability $L_\perp|\psi(\mathbf{r}_\perp/L_\perp)|^2$ (**d**) along the gray 1D chain for different $L_\perp$. Darker (lighter) color corresponds to smaller (larger) system size $L_\perp$. The inset in **d** shows the fitting results for $L_\perp = 90$. **e**, The rescaled probability along the blue 1D chain. **f**, Scaling behavior of the characteristic length $\xi_\perp$. Gray (blue) gradient corresponds to the gray (blue) 1D chain in **b**.

accumulation at the corner.

Finally, we remark that delocalized modes in a square-lattice ribbon geometry also exhibit a boundary accumulation, shown in Fig. 2c in the main text. The corresponding eigenstate profiles can be found in Fig. 5a (left). We find that these modes slightly localized at the two ends of the ribbon geometry correspond to the eigenstate numbers close to 1 and N'. Again, these modes correspond to the bulk modes in the vicinity of the light cone and, thus, can be regarded as the consequence of the long-range interaction.

2. *System-size dependence of characteristic length*

The scale-free localization of skin mode can also be observed in a finite-size 2D atomic lattice. To understand how this happens, we can start by considering a rectangular-lattice ribbon, which has a width of L_x unit cells and hosts skin modes at the boundaries in x-direction. Then let us shorten this ribbon along the infinite direction and impose open boundaries in this direction. If in this direction the number of unit cells, L_y, is large enough and the boundaries in this direction do not host skin modes, it is expected that such a finite system would still exhibit the scale-free localization.

Now we numerically identify the scaling behavior of skin modes in a 2D finite system. Along above discussions, we consider a rectangular lattice whose boundary is a parallelogram (Fig. 7b) and the skin modes only emerge at the oblique boundaries (the $(\bar{1}1)$ plane). Here we choose a skin mode at small system size ($L_x = 20$) with an energy, say E_{20}, and then find the skin mode at larger system size with the energy closest to E_{20}. The chosen mode is denoted as the blue dot in the spectrum shown in Fig. 7a. The probability distribution $|\psi(\mathbf{r})|^2$ of the chosen skin mode is shown in Fig. 7b. Therefore, we can determine the characteristic length of the skin mode along a 1D, staircase-like atomic chain with $L_\perp = L_x$ unit cells. Different choices of 1D chains are indicated by the gray and blue circles in Fig. 7b.

The normalized probability distributions $|\psi(\mathbf{r}_x)|^2$ along the blue 1D chain for different L_x are shown in Fig. 7c. It is evident that $|\psi(\mathbf{r}_x)|^2$ attenuates from both ends. Since the gray 1D chain in Fig. 7b bisects the parallelogram, we determine the characteristic length $\xi_\perp$ of the rescaled probability $L_\perp|\psi(\mathbf{r}_\perp/L_\perp)|^2$ in Figure 7d by using the fitting function $A(e^{-(\mathbf{r}_\perp-1)/(\xi_\perp/2)} + e^{-(L_\perp-\mathbf{r}_\perp)/(\xi_\perp/2)})^2$. We find that the characteristic length increases as system size increases, presenting a scale-free characteristic length at larger system size. The fitting result is shown in Fig. 7f.

Finally, to check whether the result depends on the choices of 1D chains, we consider the blue 1D chain in Fig. 7b. We can still observe the coalescence of rescaled probability distribution in Fig. 7e. To determine the characteristic length, we fit the rescaled probability distribution in the right-half chain by using the function

$Ae^{-(L_\perp - \mathbf{r}_\perp)/\xi_\perp}$. Fig. 7f shows the similar scaling behavior as that of the gray chain. Hence, the scale-free localization of skin mode can be witnessed in a finite-size 2D atomic lattice.

SUPPLEMENTARY NOTE 6. SIMULATIONS ON OPTICAL RESPONSES OF ATOMIC LATTICES

In this section, we start from the review of light scattering off a collection of point-like dipoles [6], which shows the collective optical responses and the single diffraction order from the normal process (no change in the in-plane momentum) in the 2D Brillouin zone. By eliminating the only detuning-dependent quantity, i.e., the Green's function or the collective polarizability, we are able to extract the two-band effective bulk Hamiltonian from the scattering matrices at only two detunings, and its eigenenergy spectrum reflects the emergence of EP by tuning the lattice constant ratio. This scattering method requires no detuning-resolved measurement followed by the fitting with Lorentzian functions [18] or the resonance enhancement [8], which is quite different from the existing techniques to the best of our knowledge.

1. *Scattering problem*

In the weak driven limit, each atom labeled by lattice site n in a 2D finite atomic array composed of N identical atoms driven at detuning $|\delta| \ll \omega_0 = cq$ (near resonance) can be modeled by a classical point-like dipole emitter $\mathbf{p}(\mathbf{r}'_m)$ located at $\mathbf{r}'_n$ on the 2D atomic plane ($z'_n = 0$). The scattering problem with a given incident field $\mathbf{E}_{\text{inc}}(\mathbf{r})$ is now described by the solution to the Maxwell's curl equation $\nabla \times \nabla \times \mathbf{E}(\mathbf{r}) - q^2\mathbf{E}(\mathbf{r}) = q^2\epsilon_0^{-1}\sum_{m=1}^{N}\mathbf{p}(\mathbf{r}'_m)$

$$\begin{aligned}\mathbf{E}_{\text{tot}}(\mathbf{r}) &= \mathbf{E}_{\text{inc}}(\mathbf{r}) + \frac{q^2}{\epsilon_0}\sum_{m=1}^{N}\overline{\overline{\mathbf{G}}}_0(\mathbf{r}-\mathbf{r}'_m)\cdot\mathbf{p}(\mathbf{r}'_m)\\ &= \mathbf{E}_{\text{inc}}(\mathbf{r}) + \mathbf{E}_{\text{sc}}(\mathbf{r}),\end{aligned} \tag{49}$$

$$\mathbf{p}(\mathbf{r}'_m) = \alpha(\delta)\mathbf{E}_{\text{tot}}(\mathbf{r}'_m), \tag{50}$$

where $\alpha(\delta)$ is the bare polarizability of an atom near resonance (ignore the non-radiative decay rate)

$$\alpha(\delta) = -\frac{3}{4\pi^2}\epsilon_0\lambda^3\frac{\Gamma_0/2}{\delta + i\Gamma_0/2}. \tag{51}$$

The spatial distribution of induced dipoles $\mathbf{p}(\mathbf{r}'_m)$ can be obtained from Eq. (49) by setting the field position $\mathbf{r}$ on the 2D atomic plane and excluding the self-interaction $\overline{\overline{\mathbf{G}}}_0(\mathbf{0})$. Inserting Eq. (50) into Eq. (49), we have

$$\begin{aligned}\mathbf{p} &= \left[\alpha^{-1}(\delta)\mathbb{1} - \frac{q^2}{\epsilon_0}\overline{\overline{\mathbb{G}}}_0\right]^{-1}\cdot\mathbf{E}_{\text{inc}}\\ &= -\frac{2}{3\Gamma_0}\frac{4\pi^2}{\epsilon_0\lambda^3}\frac{1}{(\delta+\omega_0)\mathbb{1} - \mathcal{H}_{\text{eff}}^{3\text{D}}/\hbar}\cdot\mathbf{E}_{\text{inc}}\\ &= \overline{\overline{\mathbb{G}}}(\delta)\cdot\mathbf{E}_{\text{inc}},\end{aligned} \tag{52}$$

where the induced dipoles $\mathbf{p} = [p_\mu(\mathbf{r}'_m)]_{3N\times1}$, the dyadic Green's function $\overline{\overline{\mathbb{G}}}_0 = [G_{0,\mu\nu}(\mathbf{r}'_{mn})]_{3N\times3N}$, the incident field $\mathbf{E}_{\text{inc}} = [E_{\text{inc},\mu}(\mathbf{r}'_m)]_{3N\times1}$, the effective Hamiltonian kernel $\mathcal{H}_{\text{eff}}^{3\text{D}}$ in Eq. (3), and the collective polarizability $\overline{\overline{\mathbb{G}}}(\delta) = [G_{\mu\nu}(\delta,\mathbf{r}'_{mn})]_{3N\times3N}$ on the atomic lattice plane are defined in the tensor product space between the atomic site and three independent polarizations. Collective polarizability $\overline{\overline{\mathbb{G}}}(\delta)$ can be also regarded as the Green's function in the linear response theory. The scattered field at $\mathbf{r}$ generated by these induced dipoles at $\mathbf{r}'$ is given by the dyadic Green's function according to Eq. (49).

$$\begin{aligned}\mathbf{E}_{\text{sc}}(\mathbf{r}) &= \frac{q^2}{\epsilon_0}\sum_{m=1}^{N}\overline{\overline{\mathbf{G}}}_0(\mathbf{r}-\mathbf{r}'_m)\cdot\mathbf{p}(\mathbf{r}'_m)\\ &= \sum_{m=1}^{N}\overline{\overline{\mathbf{g}}}_{\text{sc}}(\mathbf{r}-\mathbf{r}'_m)\cdot\sum_{n\neq m}\overline{\overline{\mathbf{G}}}(\delta,\mathbf{r}'_{mn})\cdot\mathbf{E}_{\text{inc}}(\mathbf{r}'_n)\\ &= \sum_{n=1}^{N}\overline{\overline{\mathbf{S}}}(\delta,\mathbf{r}-\mathbf{r}'_n)\cdot\mathbf{E}_{\text{inc}}(\mathbf{r}'_n),\end{aligned} \tag{53}$$

where $\overline{\overline{\mathbf{S}}}(\delta,\mathbf{r}-\mathbf{r}'_n)$, scattering matrix associated with the phase shift from the propagation, is a 3×3 matrix.

For an infinite 2D atomic array shined by an incident plane wave $\mathbf{E}_{\text{inc}}(\mathbf{r}) = \mathbf{E}_{0,\mathbf{k}}e^{i\mathbf{k}\cdot\mathbf{r}}$, the scattered field from the atomic array is now given by Eq. (53)

$$\begin{aligned}\mathbf{E}_{\text{sc}}(\mathbf{r}) &= \sum_m\overline{\overline{\mathbf{g}}}_{\text{sc}}(\mathbf{r}-\mathbf{r}'_m)\cdot\sum_{n\neq m}\overline{\overline{\mathbf{G}}}(\delta,\mathbf{r}'_{mn})\cdot\mathbf{E}_{0,\mathbf{k}}e^{i\mathbf{k}_\parallel\cdot\mathbf{r}'_n}\\ &= \overline{\overline{\mathbf{g}'}}_{\text{sc}}(\mathbf{k},\mathbf{r})\cdot\overline{\overline{\mathbf{G}'}}(\delta,\mathbf{k}_\parallel)\cdot\mathbf{E}_{0,\mathbf{k}}\\ &= \overline{\overline{\mathbf{S}'}}(\delta,\mathbf{k},\mathbf{r})\cdot\mathbf{E}_{0,\mathbf{k}},\end{aligned} \tag{54}$$

with

$$\overline{\overline{\mathbf{g}'}}_{\text{sc}}(\mathbf{k},\mathbf{r}) = \sum_m\frac{q^2}{\epsilon_0}\overline{\overline{\mathbf{G}}}_0(\mathbf{r}-\mathbf{r}'_m)e^{i\mathbf{k}_\parallel\cdot\mathbf{r}'_m}, \tag{55}$$

and

$$\overline{\overline{\mathbf{G}'}}(\delta,\mathbf{k}_\parallel) = \sum_{n\neq m}\overline{\overline{\mathbf{G}}}(\delta,\mathbf{r}'_{mn})e^{-i\mathbf{k}_\parallel\cdot\mathbf{r}'_{mn}}, \tag{56}$$

where the Fourier component $\overline{\overline{\mathbf{g}'}}_{\text{sc}}(\mathbf{k},\mathbf{r})$ is obtained from Eq. (9) by integrating out its k_z component

$$\overline{\overline{\mathbf{G}}}_0(\mathbf{r}-\mathbf{r}'_m)=\int\frac{d^2k_\parallel}{(2\pi)^3}\Bigg\{\Bigg[\overline{\overline{\mathbf{I}}}-\Big(1-\frac{\mathbf{k}_\parallel^2}{q^2}\Big)\mathbf{e}_z\otimes\mathbf{e}_z-\frac{1}{q^2}\mathbf{k}_\parallel\otimes\mathbf{k}_\parallel\Bigg]\Bigg[i\pi\Theta(q^2-\mathbf{k}_\parallel^2)\frac{e^{i\sqrt{q^2-\mathbf{k}_\parallel^2}|z|}}{\sqrt{q^2-\mathbf{k}_\parallel^2}}+\pi\Theta(\mathbf{k}_\parallel^2-q^2)\frac{e^{-\sqrt{\mathbf{k}_\parallel^2-q^2}|z|}}{\sqrt{\mathbf{k}_\parallel^2-q^2}}\Bigg]$$
$$+\Bigg[-\frac{1}{q^2}(\mathbf{k}_\parallel\otimes\mathbf{e}_z+\mathbf{e}_z\otimes\mathbf{k}_\parallel)\mathrm{sgn}(z)\Bigg]\Bigg[i\pi\Theta(q^2-\mathbf{k}_\parallel^2)e^{i\sqrt{q^2-\mathbf{k}_\parallel^2}|z|}+\pi\Theta(\mathbf{k}_\parallel^2-q^2)e^{-\sqrt{\mathbf{k}_\parallel^2-q^2}|z|}\Bigg]\Bigg\}e^{i\mathbf{k}_\parallel\cdot(\mathbf{r}-\mathbf{r}'_m)}$$
$$=\int\frac{d^2k_\parallel}{(2\pi)^2}e^{i\mathbf{k}_\parallel\cdot(\mathbf{r}-\mathbf{r}'_m)}\overline{\overline{\mathbf{g}}}_0(\mathbf{k}_\parallel,z). \tag{57}$$

Similar to Eq. (8), the Fourier component $\overline{\overline{\mathbf{g}'}}_{\mathrm{sc}}(\mathbf{k},\mathbf{r})$ can be regarded as the infinite summation on the Fourier transformed $\overline{\overline{\mathbf{g}}}_0(\mathbf{k}_\parallel,\mathbf{r})$

$$\overline{\overline{\mathbf{g}'}}_{\mathrm{sc}}(\mathbf{k},\mathbf{r})=\sum_{\mathbf{G}}\frac{q^2}{\epsilon_0}\frac{\overline{\overline{\mathbf{g}}}_0(\mathbf{G}+\mathbf{k}_\parallel,z)}{|\mathbf{a}_1\times\mathbf{a}_2|}e^{i\mathbf{k}_\parallel\cdot\mathbf{r}}. \tag{58}$$

By identifying the z dependence of Eq. (58), we can categorize the collective emission into the propagating wave $e^{i\sqrt{q^2-\mathbf{k}_\parallel^2}|z|}$ and the evanescent wave $e^{-\sqrt{\mathbf{k}_\parallel^2-q^2}|z|}$, in the same manner as what we have discussed in Eq. (9), and only the propagating wave contributes to far-field radiations. Also, the existence of $|z|$ ensures the symmetric scattered field with respect to the atomic plane (see the top panels of Fig. 8a,b). When there is only one mode ($\mathbf{G}=0$) in reciprocal space that contributes to the decay (single diffraction order when $a/\lambda<0.5$ for square lattice), the Fourier component $\overline{\overline{\mathbf{g}'}}_{\mathrm{sc}}(\mathbf{k},\mathbf{r})$ in 3D reciprocal space is

$$\overline{\overline{\mathbf{g}'}}_{\mathrm{sc}}(\mathbf{k},\mathbf{r})\big|_{q|z|\gg1}=\frac{q^2}{\epsilon_0}\frac{ie^{i\mathbf{k}_\parallel\cdot\mathbf{r}}e^{i\sqrt{q^2-\mathbf{k}_\parallel^2}|z|}}{2\,|\mathbf{a}_1\times\mathbf{a}_2|\,\sqrt{q^2-\mathbf{k}_\parallel^2}}$$
$$\times\left[\begin{array}{l}\overline{\overline{\mathbf{I}}}-\Big(1-\frac{\mathbf{k}_\parallel^2}{q^2}\Big)\mathbf{e}_z\otimes\mathbf{e}_z-\frac{1}{q^2}\mathbf{k}_\parallel\otimes\mathbf{k}_\parallel\\-\frac{1}{q^2}(\mathbf{k}_\parallel\otimes\mathbf{e}_z+\mathbf{e}_z\otimes\mathbf{k}_\parallel)\mathrm{sgn}(z)\sqrt{q^2-\mathbf{k}_\parallel^2}\end{array}\right]$$
$$=g'_{\mathrm{sc}}(\mathbf{k},\mathbf{r})\big|_{q|z|\gg1}(\overline{\overline{\mathbf{I}}}-\mathbf{e}_k\otimes\mathbf{e}_k)$$
$$=g'_{\mathrm{sc}}(\mathbf{k},\mathbf{r})\big|_{q|z|\gg1}(\mathbf{e}_p\otimes\mathbf{e}_p+\mathbf{e}_s\otimes\mathbf{e}_s), \tag{59}$$

which projects the scattered field on the subspace spanned by p- and s-polarized basis vectors due to the dyadic operator $\overline{\overline{\mathbf{I}}}+q^{-2}\nabla\otimes\nabla$ of RDDI and $k_z=\mathrm{sgn}(z)\sqrt{q^2-\mathbf{k}_\parallel^2}$. Here the p- and s-polarized basis vectors $\mathbf{e}_p=\cos\theta\cos\phi\mathbf{e}_x+\cos\theta\sin\phi\mathbf{e}_y-\sin\theta\mathbf{e}_z$ and $\mathbf{e}_s=\sin\phi\mathbf{e}_x-\cos\phi\mathbf{e}_y$ are defined for the scattered far-field plane wave indicated by a wave vector $\mathbf{k}$ with magnitude $|\mathbf{k}|=q$, the polar angle $\theta=\tan^{-1}(k_z/q)$, and azimuthal angle $\phi=\tan^{-1}(k_y/k_x)$.

2. *Extracted effective Hamiltonian*

If a 2D finite atomic array exhibits the corresponding bulk properties, we can assume $\overline{\overline{\mathbf{g}}}_{\mathrm{sc}}(\mathbf{r}-\mathbf{r}')$ also serves as a projector such that the scattering matrix $\overline{\overline{\mathbf{S}}}(\delta,\mathbf{r}-\mathbf{r}')$ on the transverse polarization subspace at each incident angle can be determined by calculating the scattered fields arising from p- and s-polarized incident fields. In this case, we are able to extract the information of the counterpart of the Green's function $\overline{\overline{\mathbf{G}}}(\delta,\mathbf{r}')$ in 2D reciprocal space (Brillouin zone) $\overline{\overline{\mathbf{G}'}}(\delta,\mathbf{k}_\parallel)$. The reason we are interested in this $\overline{\overline{\mathbf{G}'}}(\delta,\mathbf{k}_\parallel)$ is that $\overline{\overline{\mathbf{G}'}}(\delta,\mathbf{k}_\parallel)$ involves the effective Hamiltonian kernel, which is obtained from the Fourier transformation of Eq. (52)

$$\mathbf{p}_{\mathbf{k}_\parallel}=\Bigg[\alpha^{-1}(\delta)\overline{\overline{\mathbf{I}}}-\frac{q^2}{\epsilon_0}\sum_{n\neq m}\overline{\overline{\mathbf{G}_0}}(\mathbf{r}'_{mn})e^{-i\mathbf{k}_\parallel\cdot\mathbf{r}'_{mn}}\Bigg]^{-1}\cdot\mathbf{E}_{\mathrm{inc},\mathbf{k}_\parallel}$$
$$=-\frac{3}{4\pi^2}\epsilon_0\lambda^3\frac{\Gamma_0}{2}\Big[(\delta+\omega_0)\overline{\overline{\mathbf{I}}}-\frac{1}{\hbar}\mathcal{H}^{\mathrm{3D}}_{\mathrm{eff}}(\mathbf{k}_\parallel)\Big]^{-1}\cdot\mathbf{E}_{\mathrm{inc},\mathbf{k}_\parallel}$$
$$=\overline{\overline{\mathbf{G}'}}(\delta,\mathbf{k}_\parallel)\cdot\mathbf{E}_{\mathrm{inc},\mathbf{k}_\parallel}, \tag{60}$$

where $\mathcal{H}^{\mathrm{3D}}_{\mathrm{eff}}(\mathbf{k}_\parallel)$ is defined in Eq. (6). Comparing the denominators of Eq. (51) and $\overline{\overline{\mathbf{G}'}}(\delta,\mathbf{k}_\parallel)$, we can see the real and imaginary parts of $\mathcal{H}^{\mathrm{3D}}_{\mathrm{eff}}(\mathbf{k}_\parallel)$ actually represent the collective Lamb shift and the overall decay rate $E_{1,2}(\mathbf{k})=\hbar(\omega_0+\Delta_{\mathbf{k}})-\frac{i}{2}\hbar\Gamma_{\mathbf{k}}$, such that the information of EPs of a two-band effective Hamiltonian kernel $\mathcal{H}_{\mathrm{eff}}(\mathbf{k}_\parallel)$ in Eq. (30) could be extracted from the Fourier transformed Green's function $\overline{\overline{\mathbf{G}'}}(\delta,\mathbf{k}_\parallel)$ if we can discard the out-of-plane components.

Another thing to be addressed in order to extract bulk Hamiltonians on 2D atomic arrays is that the scattering matrix lives in the transverse polarization subspace, defined in 3D real space $\mathbf{r}$ spanned by $\mathbf{e}_{p,s}$, while the two-band effective Hamiltonian lives in the in-plane polarization subspace, defined on 2D atomic array $\mathbf{r}'$ spanned by $\mathbf{e}_{x,y}$. To transfer the transverse polarization subspace to the in-plane polarization subspace at single diffraction order, notice that Eq. (60) means that far-field radiations always live in the transverse polarization subspace, and $\mathcal{P}_{p,s}\overline{\overline{\mathbf{S}'}}(\delta,\mathbf{k},\mathbf{r})\big|_{q|z|\gg1}=g'_{\mathrm{sc}}(\mathbf{k},\mathbf{r})\big|_{q|z|\gg1}\mathcal{P}_{p,s}\overline{\overline{\mathbf{G}'}}(\delta,\mathbf{k}_\parallel)$ are both projected onto the

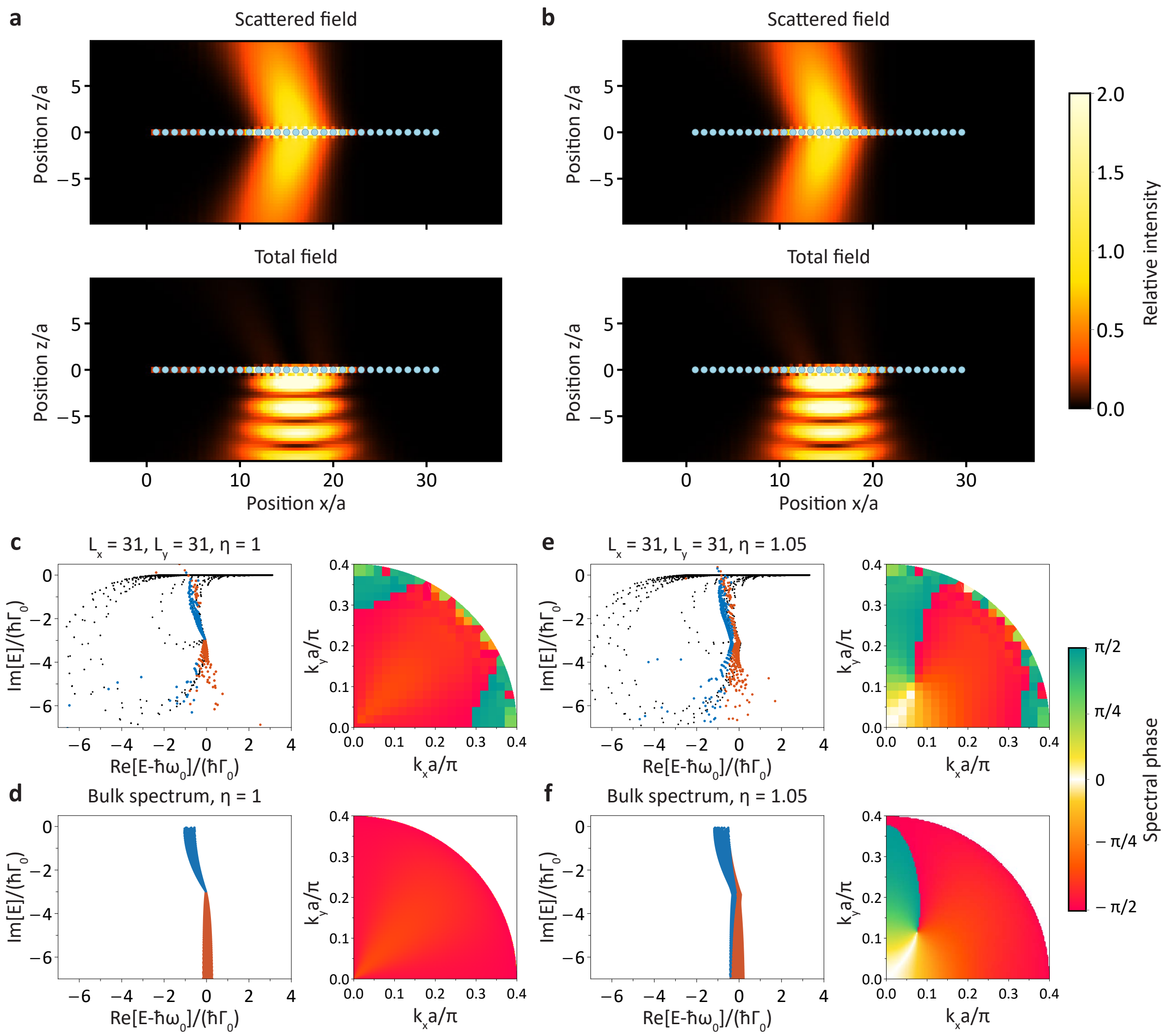

Supplementary Fig. 8. Bulk properties extracted from scattering problems in finite systems. High-reflectivity optical mirror formed by 2D square (**a**, $\eta = 1$) and rectangular (**b**, $\eta = 1.05$) subwavelength atomic lattices ($a/\lambda = 0.2$) with rectangle-shaped open boundaries. The probe beams in the simulations are p-polarized Gaussian beams with in-plane momenta $\mathbf{k}_\parallel = -0.1\pi/a\mathbf{e}_x$ (at oblique incident angle $\theta \approx 14.5°$), which propagate toward positive z direction and shine at the centers $(x, y, z) = (16a/\eta, 16a, 0a)$ of atomic lattices with beam waists $w_0 = 4a = 0.8\lambda$ and detuning $\delta = 0\Gamma_0$. Here we consider $y/a = 16$ plane to draw the relative intensity $|\mathbf{E}_{\rm sc,tot}(\mathbf{r})|^2/|\mathbf{E}_{\rm inc}(\mathbf{r})|^2$ of scattered field $\mathbf{E}_{\rm sc}(\mathbf{r})$ (top panels) and total field $\mathbf{E}_{\rm tot}(\mathbf{r})$ (bottom panels), and the blue dots represent the configuration of atoms on $y/a = 16$ plane. **c**-**f**, Eigenenergy spectra (left panels) and spectral windings of eigenenergies indicated by a spectral phase $\mathrm{Arg}[E_1(\mathbf{k}) - E_2(\mathbf{k})]$ (right panels) extracted from the simulations in finite systems (**c**,**e**, we consider $\delta = \pm 0.5\Gamma_0$ to use Eq. (63)) and obtained from bulk band structures (**d**,**f**), respectively. At small incident angles, finite system simulations in both lattice configurations capture the bulk properties well, where the spectral phase presents the half-integer spectral winding around EP, has a discontinuity at the bulk Fermi arc, and becomes zero at imaginary bulk Fermi arc in the right panel of **e**. In the numerical simulations, we consider a far-detuned π transition with detuning $30\Gamma_0$ with respect to two in-plane polarizations $|\pm\rangle$.

transverse polarization subspace. Since in-plane polarizations and out-of-plane polarization in $\overline{\overline{\mathbf{G}'}}(\delta, \mathbf{k}_\parallel)$ are decoupled due to the 2D geometry, only the pp component of $\mathcal{P}_{p,s}\overline{\overline{\mathbf{G}'}}(\delta, \mathbf{k}_\parallel)$ contains the zz component of $\overline{\overline{\mathbf{G}'}}(\delta, \mathbf{k}_\parallel)$, and it becomes negligible when out-of-plane polarization is far-detuned from in-plane polarizations or the level structure is V-type. For instance, we can express the pp component of $\mathcal{P}_{p,s}\overline{\overline{\mathbf{G}'}}(\delta, \mathbf{k}_\parallel)$ in terms of $\mathcal{P}_{\rm IP}\overline{\overline{\mathbf{G}'}}(\delta, \mathbf{k}_\parallel)$ and $\mathcal{P}_z\overline{\overline{\mathbf{G}'}}(\delta, \mathbf{k}_\parallel)$ (where IP stands for in-plane)

$$\begin{aligned}\left[\mathcal{P}_{p,s}\overline{\overline{\mathbf{G}'}}(\delta, \mathbf{k}_\parallel)\right]_{pp} &= (\mathcal{P}_{\rm IP}\mathbf{e}_p)^\dagger \cdot \left[\mathcal{P}_{\rm IP}\overline{\overline{\mathbf{G}'}}(\delta, \mathbf{k}_\parallel)\right] \cdot (\mathcal{P}_{\rm IP}\mathbf{e}_p) \\ &+ \underbrace{(\mathcal{P}_z\mathbf{e}_p)^\dagger \cdot \left[\mathcal{P}_z\overline{\overline{\mathbf{G}'}}(\delta, \mathbf{k}_\parallel)\right] \cdot (\mathcal{P}_z\mathbf{e}_p)}_{\text{negligible}},\end{aligned} \tag{61}$$

where

$$\mathcal{P}_{\mathrm{IP},z}\overline{\overline{\mathbf{G}'}}(\delta,\mathbf{k}_\parallel) = -\frac{3}{4\pi^2}\epsilon_0\lambda^3\frac{\Gamma_0}{2}\left[(\delta+\omega_0)\mathcal{P}_{\mathrm{IP},z}\overline{\overline{\mathbf{I}}}-\frac{1}{\hbar}\mathcal{P}_{\mathrm{IP},z}\mathcal{H}^{\mathrm{3D}}_{\mathrm{eff}}(\mathbf{k}_\parallel)\right]^{-1}. \quad (62)$$

In this case, $\mathcal{P}_{p,s}\overline{\overline{\mathbf{G}'}}(\delta,\mathbf{k}_\parallel)$ involves only the in-plane part of $\overline{\overline{\mathbf{G}'}}(\delta,\mathbf{k}_\parallel)$ such that we can obtain $\mathcal{P}_{\mathrm{IP}}\overline{\overline{\mathbf{G}'}}(\delta,\mathbf{k}_\parallel)$ and $\mathcal{H}_{\mathrm{eff}}(\mathbf{k}_\parallel)=\mathcal{P}_{\mathrm{IP}}\mathcal{H}^{\mathrm{3D}}_{\mathrm{eff}}(\mathbf{k}_\parallel)$ from two scattering matrices at different detunings δ_1 and δ_2. Specifically, we consider scattering matrices at two detunings δ_1 and δ_2 to eliminate their identical $\overline{\overline{\mathbf{g}'}}_{\mathrm{sc}}(\mathbf{k},\mathbf{r})\big|_{q|z|\gg1}$ parts, from which we obtain the following detuning-independent effective Hamiltonian kernel composed of only two in-plane polarizations

$$\mathcal{H}_{\mathrm{eff}}(\mathbf{k}_\parallel) = (\delta_1+\omega_0)\mathcal{P}_{\mathrm{IP}}\overline{\overline{\mathbf{I}}} - (\delta_1-\delta_2)\left[\mathcal{P}_{\mathrm{IP}}\overline{\overline{\mathbf{S}'}}(\delta_1,\mathbf{k},\mathbf{r})\big|_{q|z|\gg1}\right]^{-1}\cdot\left\{\left[\mathcal{P}_{\mathrm{IP}}\overline{\overline{\mathbf{S}'}}(\delta_1,\mathbf{k},\mathbf{r})\big|_{q|z|\gg1}\right]^{-1}-\left[\mathcal{P}_{\mathrm{IP}}\overline{\overline{\mathbf{S}'}}(\delta_2,\mathbf{k},\mathbf{r})\big|_{q|z|\gg1}\right]^{-1}\right\}^{-1}. \quad (63)$$

To demonstrate that a 2D finite atomic array can exhibit the bulk properties, we use Eq. (63) to extract and estimate the bulk Hamiltonian from the scattering problem of a 2D finite atomic array. In Fig. 8a,b, we consider the scattering problems of 2D finite square and rectangular atomic lattices with rectangle-shaped open boundaries shined by an oblique incident Gaussian beam towards the positive z direction. We calculate the ratio between the scattered field and the incident field at $z>0$ region at the intersection between the central line of incident field and $z=5a$ plane in the far-field region ($q|z|=10\pi a/\lambda=5\pi\gg1$) to discard the evanescent wave corresponding to the subradiant modes. The reason we choose the fields at the same point is to eliminate the traveling phase in $\overline{\overline{\mathbf{S}}}(\delta,\mathbf{r}-\mathbf{r}')$. By treating the far-field part of $\overline{\overline{\mathbf{S}}}(\delta,\mathbf{r})$ obtained from the finite-size simulation as the far-field part of $\overline{\overline{\mathbf{S}}}(\delta,\mathbf{k},\mathbf{r})$ in the infinite system, where $\mathbf{k}$ corresponds to the same oblique incident angle, we are able to use Eq. (63) to extract and estimate the bulk Hamiltonian $\mathcal{H}_{\mathrm{eff}}(\mathbf{k}_\parallel)=\mathcal{P}_{\mathrm{IP}}\mathcal{H}^{\mathrm{3D}}_{\mathrm{eff}}(\mathbf{k}_\parallel)$ since both $\overline{\overline{\mathbf{S}}}(\delta,\mathbf{r})$ and $\overline{\overline{\mathbf{S}'}}(\delta,\mathbf{k},\mathbf{r})$ are 3×3 matrices and describe the relation between the scattered and incident field. Note that the in-plane momentum here always lies within the light cone since the in-plane momentum provided by a resonant light is bounded by the magnitude of wave vector $q=2\pi/\lambda$.

The main reason we can use Eq. (63) to extract and estimate the bulk Hamiltonian from the scattering matrices is that the dyadic Green's function $\overline{\overline{\mathbf{g}}}_{\mathrm{sc}}(\mathbf{k},\mathbf{r})$ is approximately detuning independent when the detuning δ is not too large compared to the resonant frequency $\omega_0=cq\gg|\delta|$ (near resonance). Once $\overline{\overline{\mathbf{g}}}_{\mathrm{sc}}(\mathbf{k},\mathbf{r})$ depends on the detuning δ, we are not able to easily eliminate the $\overline{\overline{\mathbf{g}}}_{\mathrm{sc}}(\mathbf{k},\mathbf{r})$ part in the scattering matrix to extract the effective Hamiltonian $\mathcal{H}_{\mathrm{eff}}(\mathbf{k}_\parallel)=\mathcal{P}_{\mathrm{IP}}\mathcal{H}^{\mathrm{3D}}_{\mathrm{eff}}(\mathbf{k}_\parallel)$. However, the collective optical response in Eq. (60) shows that the atomic array becomes transparent to the incoming light at large detuning since the dispersion shifts in the left panels of Fig. 8c-f are small. That is, we conclude that Eq. (63) works well for the 2D atomic array with photon-mediated dipole-dipole interaction. We note that the scattering matrices in Eq. (63) can be obtained in both the transmitted and reflected fields.

3. Non-Hermitian topological invariant: vorticity

With a two-band effective Hamiltonian, we can directly calculate the non-Hermitian topological invariant of its non-Hermitian degeneracy point, called the vorticity or spectral winding [18–21]

$$v=-\oint_C\frac{d\mathbf{k}}{2\pi}\cdot\nabla_{\mathbf{k}}\arg[E_1(\mathbf{k})-E_2(\mathbf{k})], \quad (64)$$

where C is a closed loop (counterclockwise) in the 2D Brillouin zone and spectral phase $\arg[E_1(\mathbf{k})-E_2(\mathbf{k})]$ is well-defined except at non-Hermitian degeneracy point. In the right panels of Fig. 8f, there is a $-\pi$ spectral phase acquired around the loop C enclosed the EP associated with a discontinuity at the bulk Fermi arc and a zero spectral phase at imaginary bulk Fermi arc, which manifests the existence of EP. This nonzero spectral phase change around the loop C enclosed the EP also reflects that EP is a branch point singularity, and such a singular point as some point $\mathbf{k}$ means that $|E_1(\mathbf{k})-E_2(\mathbf{k})|$ is zero, i.e., a degeneracy point. We note that here the dispersive bulk Fermi arc is not truncated by the light cone since the change in the lattice constant ratio η is small. Comparing the spectral phases of atomic lattices with $\eta=1$, 1.05, and 1.1 (in the main text), we can see the way EPs and dispersive Fermi arcs evolve as η changes.

In Fig. 8c,d, both the eigenenergy spectrum and the spectral phase deviate from those in an infinite system at large oblique incidence due to the diffraction from the edge. At a small incident angle, our simulation results show that a 2D finite atomic array exhibits its bulk properties and behaves like a mirror at cooperative resonance when the incident light shines in the vicinity of the center of arrays. We also check that smaller 2D finite atomic

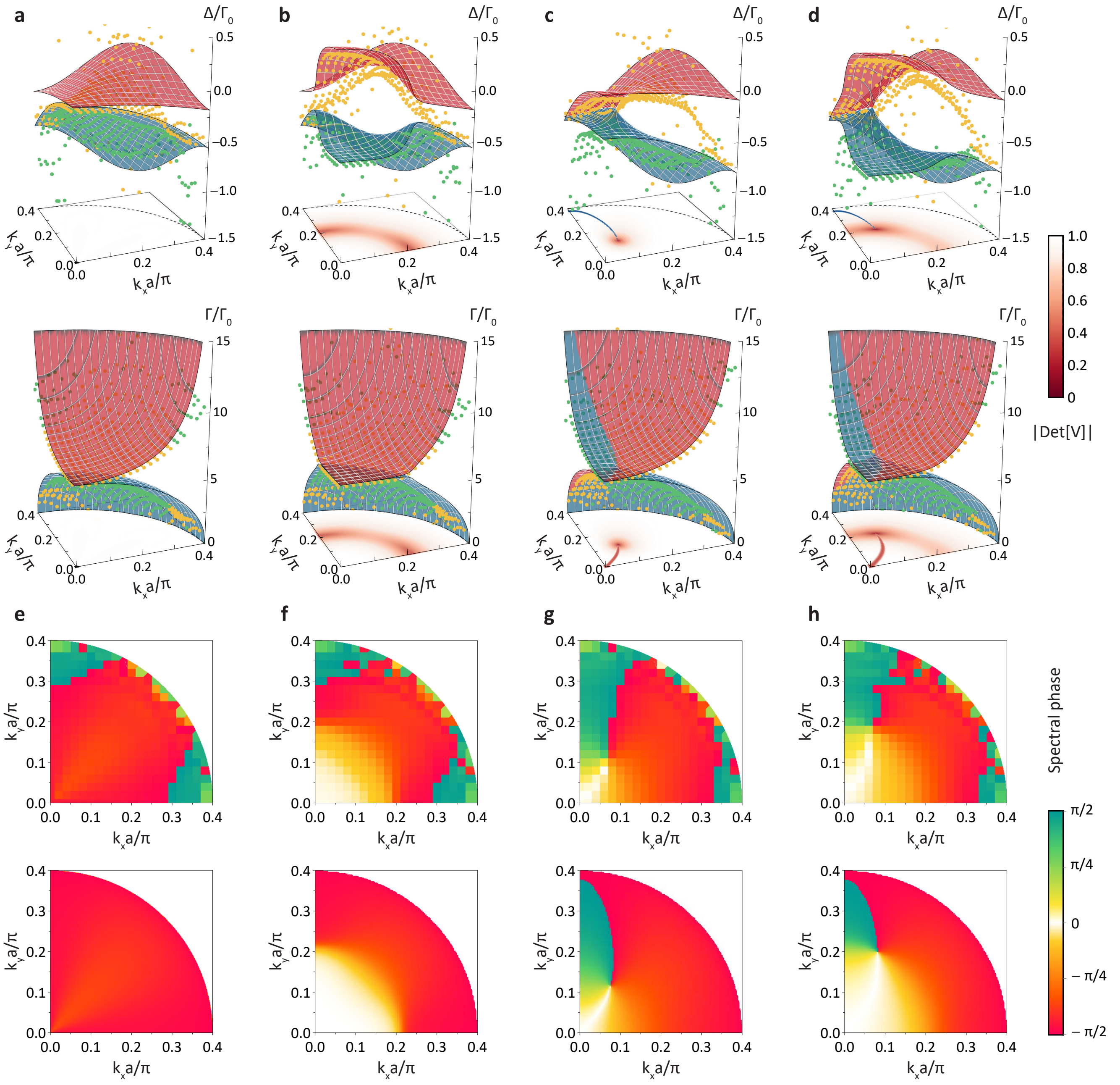

Supplementary Fig. 9. **Topologically stable exceptional points. a-d**, Collective frequency shift $\Delta_\mathbf{k}$ (top) and overall decay rate $\Gamma_\mathbf{k}$ (bottom) of infinite square (**a**,**b**, $\eta = 1$) and rectangular (**c**,**d**, $\eta = 1.05$) lattices with in-plane polarization within the light cone (black dashed circle). Two bulk energy bands $E_{1,2}(\mathbf{k}) = \hbar(\omega_0 + \Delta_\mathbf{k}) - \frac{i}{2}\hbar\Gamma_\mathbf{k}$ obtained from $\mathcal{H}_{\text{eff}}(\mathbf{k}_\parallel)$ are colored in red and blue, and those extracted from the simulations in finite systems are denoted by yellow and green dots. By applying an out-of-plane magnetic field $B\mathbf{e}_z$ with $\mu B = 0.5\Gamma_0$ in **b**,**d**, NDP in **a** is lifted by a $2\hbar\mu|B|$ shift in its energy difference between two bands. However, EPs in **c** still exist and are shifted within the light cone. **e-h**, Spectral phases $\text{Arg}[E_1(\mathbf{k}) - E_2(\mathbf{k})]$ extracted from the simulations in finite systems (top) and obtained from bulk band structures (bottom) in atomic lattices with the same lattice configuration and external magnetic field in **a-d**. All simulation parameters in **a-h**, except for the magnetic fields, are the same as Fig. 8.

arrays (14×14) show similar spectral phases as Fig. 8c,e.

Spectral phases in Fig. 8c,e also help us directly identify the vorticity of each non-Hermitian degeneracy point in square and rectangular lattices. According to the mirror symmetries with respect to the k_x and k_y axes, we can construct the full spectral phases over Brillouin zone within the light cone, and the vorticity corresponding to NDP at the origin in the right panel of Fig. 8c is zero. In contrast, the vorticity of the EP in the first quadrant of Brillouin zone in Fig. 8e is 1/2, and the vorticity of other

mirror reflected EPs are $\pm 1/2$ according to the required number of mirror reflections with respect to the EP in the first quadrant.

For the stability of these non-Hermitian degeneracy points, we can consider a nonzero out-of-plane magnetic field $B\mathbf{e}_z$ to see that NDP would be lifted (Fig. 9a,b) due to the broken Hermitian-conjugated time-reversal symmetry (Eq. 33) while EPs still exist (Fig. 9c,d). In Fig. 9b, only the real part of bulk eigenenergies are lifted since the degeneracy is determined by the discriminant $\hbar\sqrt{\mu^2 B^2 + \Gamma_0^2 \kappa_{+-}(\mathbf{k})\kappa_{-+}(\mathbf{k})}$ of the effective Hamiltonian, where magnetic field results in a $2\hbar\mu|B|$ difference in two bulk eigenenergies. Since the real part of bulk eigenenergies in the top panel of Fig. 9b are two non-intersecting bands, there is no other non-Hermitian degeneracy point within the light cone. The topological stability of EP against a weak real-valued $\hbar\mu B\sigma_z$ term arises from a nontrivial vorticity (Fig. 9g,h). That is, unless $\hbar\mu B\sigma_z$ modifies the range of the discriminant of the effective Hamiltonian significantly, the positions of EPs within the light cone could be shifted, while EPs still persist.

---

[1] Perczel, J. *et al.* Photonic band structure of two-dimensional atomic lattices. *Phys. Rev. A* **96**, 063801 (2017).

[2] Zhen, Y.-R., Fung, K.-H. & Chan, C. T. Collective plasmonic modes in two-dimensional periodic arrays of metal nanoparticles. *Phys. Rev. B* **78**, 035419 (2008).

[3] Lehmberg, R. H. Radiation from an N-Atom System. I. General Formalism. *Phys. Rev. A* **2**, 883–888 (1970).

[4] Perczel, J. *et al.* Topological quantum optics in two-dimensional atomic arrays. *Phys. Rev. Lett.* **119**, 023603 (2017).

[5] Abramowitz, M. & Stegun, I. A. (eds) *Handbook of Mathematical Functions with Formulas, Graphs, and Mathematical Tables*, 9th printing edn (New York: Dover, 1972).

[6] Shahmoon, E., Wild, D. S., Lukin, M. D. & Yelin, S. F. Cooperative resonances in light scattering from two-dimensional atomic arrays. *Phys. Rev. Lett.* **118**, 113601 (2017).

[7] Javanainen, J. & Rajapakse, R. Light propagation in systems involving two-dimensional atomic lattices. *Phys. Rev. A* **100**, 013616 (2019).

[8] Zhou, H. *et al.* Observation of bulk Fermi arc and polarization half charge from paired exceptional points. *Science* **359**, 1009–1012 (2018).

[9] Yang, Z., Schnyder, A. P., Hu, J. & Chiu, C.-K. Fermion doubling theorems in two-dimensional non-Hermitian systems for Fermi points and exceptional points. *Phys. Rev. Lett.* **126**, 086401 (2021).

[10] Kawabata, K., Shiozaki, K., Ueda, M. & Sato, M. Symmetry and topology in non-Hermitian physics. *Phys. Rev. X* **9**, 041015 (2019).

[11] Yao, S. & Wang, Z. Edge states and topological invariants of non-Hermitian systems. *Phys. Rev. Lett.* **121**, 086803 (2018).

[12] Yokomizo, K. & Murakami, S. Non-Bloch band theory of non-Hermitian systems. *Phys. Rev. Lett.* **123**, 066404 (2019).

[13] Kawabata, K., Okuma, N. & Sato, M. Non-Bloch band theory of non-Hermitian Hamiltonians in the symplectic class. *Phys. Rev. B* **101**, 195147 (2020).

[14] Li, L., Lee, C. H., Mu, S. & Gong, J. Critical non-Hermitian skin effect. *Nat. Commun.* **11**, 5491 (2020).

[15] Yokomizo, K. & Murakami, S. Scaling rule for the critical non-Hermitian skin effect. *Phys. Rev. B* **104**, 165117 (2021).

[16] Lee, C. H., Li, L., & Gong, J. Hybrid higher-order skin-topological modes in nonreciprocal systems. *Phys. Rev. Lett.* **123**, 016805 (2019).

[17] Kawabata, K., Sato, M., & Shiozaki, K. Higher-order non-hermitian skin effect. *Phys. Rev. B* **102**, 205118 (2020).

[18] Su, R. *et al.* Direct measurement of a non-Hermitian topological invariant in a hybrid light-matter system. *Sci. Adv.* **7**, eabj8905 (2021).

[19] Leykam, D., Bliokh, K. Y., Huang, C., Chong, Y. D. & Nori, F. Edge modes, degeneracies, and topological numbers in non-Hermitian systems. *Phys. Rev. Lett.* **118**, 040401 (2017).

[20] Gong, Z. *et al.* Topological phases of non-Hermitian systems. *Phys. Rev. X* **8**, 031079 (2018).

[21] Shen, H., Zhen, B. & Fu, L. Topological band theory for non-Hermitian hamiltonians. *Phys. Rev. Lett.* **120**, 146402 (2018).
